# Prevalence and Cardiopulmonary Characteristics of Post-COVID Syndrome at a Hungarian Tertiary Referral Hospital

**DOI:** 10.3390/jcm14082604

**Published:** 2025-04-10

**Authors:** Krisztina Fekete, Barbara Sándor, Anita Kacsó, Anita Pálfi, Szilard Tóth, László Deres, Eszter Szabados, László Czopf, Miklós Rábai, Roland Gál, Tamas Alexy, Tamás Habon, Kálmán Tóth, Hussain Alizadeh, Szilvia Soós, Róbert Halmosi

**Affiliations:** 1 Division of Cardiology, First Department of Medicine, Medical School, University of Pécs, 7624 Pécs, Hungary; fekete.krisztina@pte.hu (K.F.); toth.szilard@pte.hu (S.T.); deres.laszlo@pte.hu (L.D.); czopf.laszlo@pte.hu (L.C.); rabai.miklos@pte.hu (M.R.); gal.roland@pte.hu (R.G.); alexy001@umn.edu (T.A.); habon.tamas@pte.hu (T.H.); toth.kalman@pte.hu (K.T.); 2 Szentágothai Research Centre, University of Pécs, 7624 Pécs, Hungary; 3 Division of Cardiovascular Prevention and Rehabilitation, First Department of Medicine, Medical School, University of Pécs, 7623 Pécs, Hungary; sandor.barbara@pte.hu (B.S.); palfi.anita@pte.hu (A.P.); szabados.eszter@pte.hu (E.S.); 4 Division of Pulmonology, First Department of Medicine, Medical School, University of Pécs, 7623 Pécs, Hungary; kacso.anita@pte.hu (A.K.); soos.szilvia@pte.hu (S.S.); 5 Department of Medicine, Division of Cardiology, University of Minnesota, Minneapolis, MN 55455, USA; 6 Division of Haematology, First Department of Medicine, Medical School, University of Pécs, 7624 Pécs, Hungary; alizadeh.hussain@pte.hu

**Keywords:** COVID-19, complication, post-COVID, cardiopulmonary consequences

## Abstract

**Background:** After an acute COVID-19 infection, many patients suffered from various complaints called as post-COVID syndrome. **Methods:** Our post-COVID outpatient department was operational for 19 months, where patients (n = 252) underwent a detailed cardiopulmonary examination within a 3-month-long follow-up period. **Results:** Most patients (69.9%) had mild acute symptoms with a higher hospitalization risk with preexisting hypertension (*p* < 0.05) and diabetes (*p* < 0.001). Most common post-COVID symptoms were fatigue (29.4%) and dyspnea (19.1%). Echocardiographic parameters showed no abnormalities and did not change during the follow-up period. Exercise capacity was also generally normal with no change over time; however, 9.9% of patients showed significant desaturation during a 6 min walk test. This finding showed correlation (*p* < 0.01) with decreased diffusion capacity (DLCO). Generally, DLCO improved slightly but significantly (*p* < 0.05) by the end of the follow-up period (from 72.4% to 74.1%). Our key finding was a 10× higher prevalence (24.6%) of lupus anticoagulant positivity among post-COVID patients compared to the normal population (estimated at 2–4%). **Conclusions:** In conclusion, post-COVID syndrome is a common consequence even after a mild infection. Severe infections tend to lead to worse cardiopulmonary outcomes. Higher prevalence of lupus anticoagulant positivity may underline the importance of autoimmunity in the pathomechanism of post-COVID syndrome.

## 1. Introduction

The SARS-CoV-2 virus has infected at least 700 million individuals between 2020 and 2023 worldwide and, according to the Worldometer, it was associated with more than 7 million fatalities [1]. However, the Institute for Health Metrics and Evaluation (IHME) estimates that the total number of deaths attributable to the COVID-19 pandemic was even higher, closer to 19 million [2]. There is a considerable discrepancy across countries regarding the reported mortality figures highlighting the uncertainty pertinent to the true case fatality rates. This variation may potentially be attributed to differences in testing practices and the specific criteria employed by various nations to adjudicate COVID-19-attributable deaths [3]. In addition to mortality, the most severe complications secondary to SARS-CoV-2 infection include those affecting the cardiovascular, pulmonary, and immune systems. These are largely precipitated by the upper airway infection-induced constellation of cytokine storm, endothelial inflammation, dysfunction, and hypercoagulability (CAC) [4]. While the precise pathomechanism and the underlying causes of the highly variable disease presentation remain unknown, certain prognostic markers, such as serum ferritin, interleukin-6, high sensitivity CRP (hs-CRP), and D-dimer, have been identified to potentially assist in risk stratification pertinent to the initial infection [5].

Approximately 50% of patients develop a variety of persistent somatic symptoms after recovering from the acute COVID-19 infection. Various terms are used to describe this heterogenous condition, including “long COVID”, “post-acute sequelae of COVID-19”, “post-COVID-19 syndrome”, “post-COVID conditions”, and “post-acute COVID-19 syndrome (PACS)” [6,7,8]. For the purposes of this manuscript, the term “post-COVID” will be employed. Despite its high incidence, the precise underlying pathophysiological mechanisms remain poorly understood, although several potential mechanisms have been proposed. These include long-standing viral presence, persistent immune dysregulation, microbiota disruption, the development of autoimmunity, inappropriate activation of the coagulation system, endothelial dysfunction, and impaired neurological signaling [9,10]. The most common patient-reported symptom is fatigue; however, other manifestations such as dyspnea, cough, chest pain, headache, cognitive or mental decline, and olfactory dysfunction are also common [6,11,12].

Recognizing the significance of post-COVID syndrome, in 2021, the Hungarian government mandated the establishment of dedicated post-COVID outpatient clinics in regional centers with simultaneous multidisciplinary assessment. The primary objectives of this initiative were to assess the baseline characteristics of patients with post-COVID, investigate factors influencing severity of both the acute infection as well as the subsequent post-COVID syndrome, and to identify factors with potential pathogenetic or prognostic importance.

## 2. Materials and Methods

This was a single center, retrospective chart review performed in a large academic medical center that was approved by the Regional Ethical Committee of the University of Pécs (9184–PTE 2022). In response to the Hungarian Government mandate, we have initiated a specialized, multidisciplinary outpatient clinic at the 1st Department of Medicine, Clinical Center, University of Pécs, that was operational between 2 May 2021, and 31 December 2022. Patients were referred to us by general practitioners (GPs) based on symptoms that persisted at least 4 weeks after verified acute SARS-CoV-2 infection. Further inclusion criteria included: (1) age > 18 years; (2) written consent for the clinical examination and data collection, including the option for retrospective review, data analysis, and de-identified reporting in aggregate; and (3) COVID-19 infection verified by an approved rt-PCR, rapid antigen test, or IgM/IgG rapid antibody test. No specific exclusion criteria were employed.

Following informed consent, all patients underwent a standardized, comprehensive cardiopulmonary evaluation simultaneously by a cardiologist and a pulmonologist with additional consultation from experts in hematology, angiology, or endocrinology on an as-needed basis. Treatments and interventions were rendered at the time of the visit based on the results and clinical necessity. In addition to the initial visit, all individuals were scheduled for return appointments 3, 6, and 12 months after the baseline assessment.

The following baseline characteristics were collected and analyzed in this study: blood pressure, heart rate, height, body weight, body mass index (BMI), smoking history (in pack-years), history of diabetes mellitus (DM), hypertension (HT), chronic obstructive pulmonary disease (COPD), bronchial asthma, and history of any thromboembolic event (deep vein thrombosis and/or pulmonary embolism). In addition, the patients’ quality of life was assessed on a visual analogue scale (0 to 100 range).

With regards to the acute COVID-19 infection, we recorded the date and severity of initial symptom development as well as the time until clinic presentation (in weeks). Patients were divided into four groups based on their infection severity and medical treatment received: (1) mild symptoms treated at home; (2) hospitalized without respiratory failure (moderate severity); (3) hospitalized with respiratory failure (severe); and (4) critical condition requiring management in the intensive care unit (ICU). Duration of total hospitalization and the number of ICU days were collected where applicable.

During each post-COVID clinic visit, a routine 12-lead electrocardiogram (ECG) was completed. In addition, transthoracic echocardiography (TTE) was performed using the GE Vivid E9 system (GE Healthcare, Maple Grove, MN, USA) according to the European Society of Cardiology (ESC) guidelines [13]. We measured posterior and septal wall thickness, as well as the inner diameter of the left ventricle both in systole (LVIDs) and diastole (LVIDd) to assess left ventricular ejection fraction (LVEF). We measured mitral early (E) and late (A) ventricular filling velocities with pulse wave Doppler, and lateral mitral annular velocity during the early (E’) and late (A’) ventricular filling phases with tissue Doppler.

Chest X-ray was completed for all patients and was reviewed by a radiologist as well as a board-certified pulmonologist. Pulmonary function test was performed using a Vyntus Body Plethysmograph (Vyaire Medical, Irvine, CA, USA) according to the guidelines of the European Respiratory Society (ERS) [14]. Forced vital capacity (FVC), forced expiratory volume in the first second (FEV1), Tiffeneau index (FVC/FEV1), and diffusion lung capacity (DLCO) were assessed. Patients also completed a 6 min walk test (6MWT) to assess their submaximal exercise capacity according to standard clinical practice guidelines [15]. Age- and sex-adjusted normal values were established using an online calculator [16]. The completed distance of each participant was expressed as a percentage of the expected distance. A continuous pulse oximetry probe attached to the index finger during the 6MWT was used to detect any desaturation events (defined as arterial oxygen saturation falling below 90%).

In addition to the physical assessment, laboratory analysis was performed for every patient by the accredited central laboratory of the Clinical Center, University of Pécs, utilizing standard methods. This included complete blood count, comprehensive metabolic panel, ferritin, hs-CRP, D-dimer, cardiac biomarkers (high sensitivity troponin-T, N-terminal proBNP), lupus anticoagulant, and interleukin-6 and 33.

There was significant interest in attending our clinic within the initial 3 months of opening, yielding an average appointment backlog of 1 month. This has declined significantly subsequently. Following the first visit, follow-up appointments were scheduled at 3, 6, and 12 months. Owing to the fact that most patients had significant improvement or resolution in their post-COVID symptoms by the second visit, a large proportion cancelled follow-up evaluations. Therefore, we only analyzed data that are available from the baseline and 3-months appointments.

For statistical analysis, first, data were analyzed for normality with the Shapiro–Wilk test, and the Levene test was used for variance homogeneity. Statistical comparisons between the two groups were made using Student’s *t*-test or Mann–Whitney U test for non-parametric data. To compare the frequency of characteristic variables, Chi-squared or Fisher’s exact tests were used. Unless otherwise stated, values are given as mean ± SEM or as median with interquartile ranges (Q1–Q3). We used a confidence interval of 95%. Statistical analyses were performed in GraphPad Prism 9.5.1.

## 3. Results

### 3.1. Population Characteristics

A total of 252 patients were enrolled who completed their initial visit at our post-COVID clinic. Of these, 97 (38.5%) returned for a follow-up appointment at 3 months. Since, for 6 months follow-up only 13.5% of patients attended, this sample size was too small to obtain statistically considerable results; therefore, the data generated here were excluded from the analysis. Due to the limited data from 6-month follow-up, we did not collect further data for 1 year. The mean age of the cohort was 52 ± 1 years and 58.3% were female. The average BMI was 29.0 ± 0.5 without any significant sex differences. Only 29 patients (11.5%) were current smokers with an average of 5.5 ± 0.7 pack-year history. Comorbidities and their association with acute COVID-19 severity are shown in Table 1. We found that only preexisting diabetes mellitus (*p* = 0.012, OR: 2.42) and hypertension (*p* < 0.001, OR: 2.82) were significantly associated with the need for hospitalization during the acute infection, suggesting higher disease severity in these individuals.

The average time between the acute infection and first clinic appointment was 4.6 ± 0.1 months. Of those who have been referred, 69.9% had mild acute symptoms, 5.2% required hospitalization without respiratory failure, 19.9% developed respiratory failure, and 5.2% were in critical condition. The average length of ICU and total hospital stays were 10.8 ± 2.3 and 12.3 ± 1.1 days, respectively. A total of 4.7% of the patients had a thromboembolic event during the acute COVID-19 infection.

### 3.2. Post-COVID Symptoms

As Table 2 shows, the most frequent complaints consistent with post-COVID symptoms were fatigue (29.4%), dyspnea (19.0%), chest pain (10.7%), and dry cough (10.7%). Generally, patients reported a slight limitation in their average daily activities with a median quality-of-life score of 70 (60–85) at the baseline, which improved significantly (*p* < 0.001) by the 3 month follow-up, reaching a score of 80 (70–85). It is important to emphasize that only 38.5% of the cohort returned for this visit, presumably those with at least some residual symptoms, and thus it is possible that the median quality-of-life score would even be higher if follow-up data from all 252 individuals were available.

### 3.3. Clinical Testing

Initially, the median heart rate of patients on ECG was 72/min (64–81), which decreased significantly (*p* < 0.001) to 66/min (61–77) during the follow-up period. Other parameters were within the normal range on both visits without significant change.

TTE results are detailed in Table 3. Left ventricular end-diastolic and end-systolic diameters, LVEF, and diastolic parameters were within the normal range both at baseline and at month 3 with no significant change in any of the parameters evaluated.

**Figure 1:**
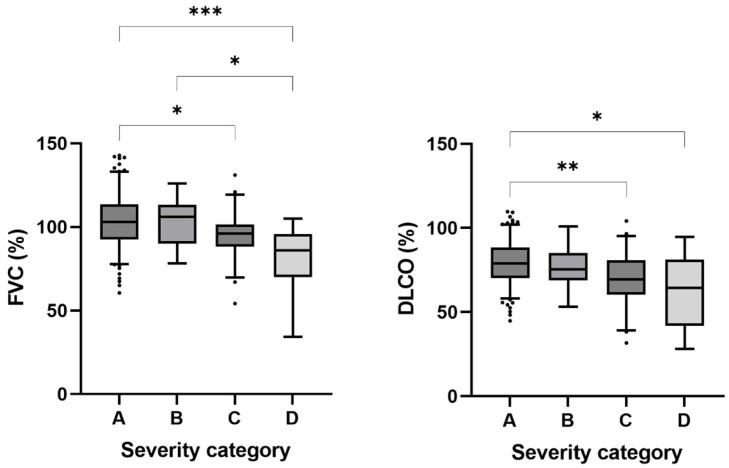
Post-COVID DLCO% and FVC% values were lower in patients with more severe acute COVID-19 infection. A: Treated at home; B: hospitalized without respiratory failure; C: hospitalized with respiratory failure; D: required intensive care; FVC: forced lung capacity; DLCO: diffusion lung capacity; *: *p* < 0.05; **: *p* < 0.01; ***: *p* < 0.001.

Pulmonary function test results at baseline and at 3 months are depicted in Table 3. Most (91.7%) had normal FVC values, while it was mildly, moderately, or severely reduced in 5.4%, 2.5%, and 0.4% of patients, respectively. Similarly, FEV1 test results were normal, mildly, or moderately reduced in 88.8%, 10.4%, and 0.83% of participants, respectively. No individuals had an FEV1 below 30% of the expected value. The Tiffeneau-index was normal in 57.7%, while it was moderately or severely reduced in 39.4% and 2.9%, respectively. Median values for all parameters remained essentially unchanged between the initial and the 3-month visits. DLCO requires special attention. At baseline, it was considered normal only in 44.0% of the patients, while it was mildly, moderately, or severely reduced in 44.4%, 8.3%, and 3.3%, respectively. Median DLCO value improved significantly over the study period from 72.4% (64.1–85.5) to 74.1% (66.3–86.0); *p* = 0.028. Based on these results, inhalation therapy (primarily ciclesonide or budesonide/formoterol) has been prescribed for 93 patients. Both FVC and DLCO values were significantly lower at baseline in patients who had severe and critical COVID-19 infection when compared to those with mild symptoms (Figure 1).

**Figure 2:**
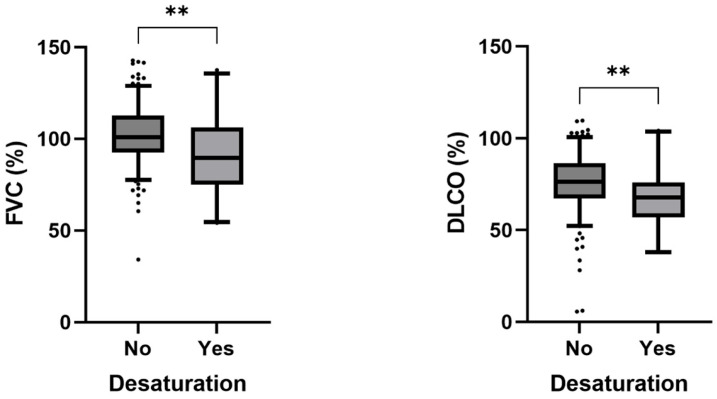
People with desaturation on 6MWT had significantly worse FVC and DLCO values on pulmonary function testing. FVC: forced vital capacity; DLCO: diffusion lung capacity; **: *p* < 0.01.

In general, our patients achieved normal or only slightly reduced 6 min walking distance at baseline (76% (65–88) of expected). Their performance remained essentially unchanged at the follow-up testing (77% (66–85) of expected). Only 25 patients (9.9%) had any desaturation events during 6MWT. This group had significantly worse DLCO and FVC values when compared to the rest of the cohort (*p* = 0.004) (see Figure 2).

### 3.4. Laboratory Testing

Laboratory test results at the initial and 3 months visits are detailed in Table 4. Although median values were essentially within the normal range on both testing dates, d-dimer, ferritin, and hs-troponin-T decreased significantly by the follow-up visit. Mirroring these changes, the proportion of patients with abnormally elevated d-dimer, ferritin, and hs-troponin-T values decreased by 3 months. Interestingly, IL-6 and IL-33 values were within the normal range on both occasions, and no relevant changes were noted in any of the other labs tested. It is important to note that the time between acute COVID-19 infection and initial laboratory testing at the post-COVID clinic was variable, maybe up to a month for some patients. It is possible that for some patients, laboratory parameters had already normalized by the time of this initial testing. With regards to lupus anticoagulant (LA), it was positive (normalized ratio cut-off over 1.2) in 24.60% of all cases, which is significantly higher than the expected 2–4% in the general population (*p* < 0.001). In addition, LA positivity was present in 41.7% of patients with a thromboembolic event during acute COVID-19 infection, or after it (5/12). In post-COVID patients, without venous thromboembolic complication, the LA positivity was 23.8% (57/240). However, this difference was not significant (*p*: 0.18).

### 3.5. Length of Hospital Stay

**Figure 3:**
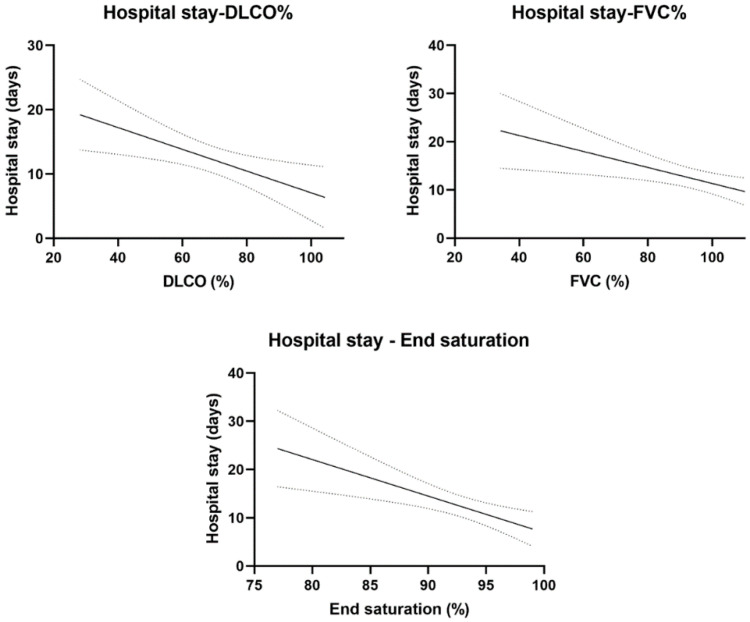
Length of hospital stay had a significant negative correlation with both FVC and DLCO values and end-test saturation during 6 min walk test. Participants with longer hospitalization during acute COVID-19 infection had a significantly lower DLCO, FVC on pulmonary function test, and end-test saturation on 6MWT. Solid lines represent the means of DLCO, FVC and end-test saturation on 6MWT. Dotted lines represent the error of mean.

Among patients who required hospitalization for COVID-19 treatment, the length of stay showed a significant negative correlation with DLCO (r^2^: 0.10; *p* = 0.008), FVC (r^2^: 0.09; *p* = 0.010) as well as oxygen saturation as measured at the end of the 6MWT (r^2^: 0.14; *p* = 0.002) (see Figure 3).

**Figure 4:**
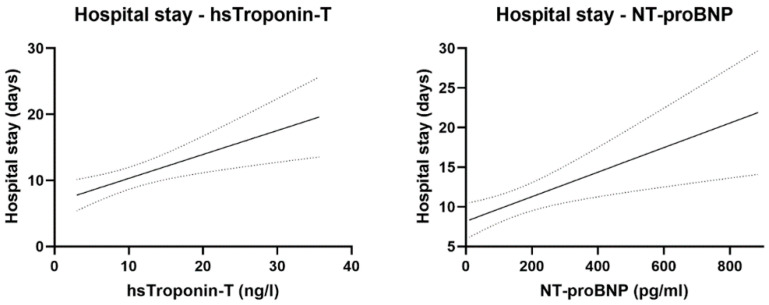
Correlation between cardiac biomarkers (hs-troponin-T and NT-proBNP) and the length of hospital stay. Previous longer COVID-19 hospitalizations resulted in significantly higher cardiac enzyme levels. Solid lines represent the means of hs-torponin-T and NT-proBNP. Dotted lines represent the error of mean.

Conversely, it showed a positive correlation with the level of cardiac biomarkers, including hs-troponin-T (r^2^: 0.13; *p* < 0.001) and NT-proBNP (r^2^: 0.12; *p* = 0.004) (see Figure 4).

**Figure 5:**
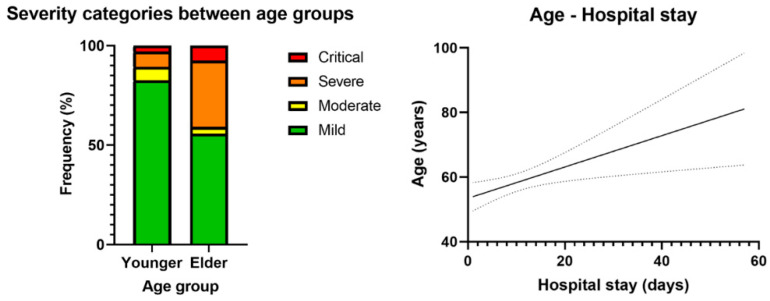
Distribution of the severity of acute infection between the two age groups and positive correlation between age and hospital stay. Participants over 52 years have had significantly more severe acute infections with almost half of them requiring hospitalization and with a longer hospital stay. The solid line represents the mean of age. Dotted lines represent the error of mean.

Finally, we divided the population into two groups based on the median age (52 years) and assessed any potential differences between the cohorts. We found a highly significant difference in the prevalence of acute infection disease severity (*p* < 0.001): The need for hospitalization reached almost 50% in those above 52 years of age, yet it was only 20% in the younger group. In addition, older individuals spent a significantly longer time in the hospital to recover (*p* = 0.011) (see Table 5 and Figure 5).

## 4. Discussion

Our study evaluated the clinical characteristics of 252 patients referred to our highly specialized outpatient clinic for suffering from post-COVID syndrome. Individuals underwent an initial assessment as well as a follow-up visit 3 months later. Overall, 30.1% required hospitalization for an average of 12.3 ± 1.1 days, with preexisting hypertension and diabetes mellitus significantly more common in these patients. Interestingly, we were not able to demonstrate a correlation between several other potential risk factors, such as pulmonary (e.g., COPD or asthma) or cardiovascular diseases, and the severity of the acute SARS-CoV-2 infection. The most common post-COVID symptoms included persistent fatigue and dyspnea, consistent with result of previous studies and surveys [6,17]. Both complaints improved significantly by the visit at 3 months. TTE parameters, including LV function, were essentially normal initially and on follow-up. Not surprising in the setting of a viral respiratory infection, DLCO was at least mildly reduced in over half of the patients initially but improved significantly, often in response to inhalation therapy, by 3 months. While interleukin levels were within the normal range both initially and on follow-up, d-dimer, ferritin, and hs-troponin-T levels decreased significantly during the study period, likely representing subsiding inflammatory changes. Older age was a significant risk factor for both disease severity and length of hospital stay.

Post-COVID syndrome is a common complication of SARS-CoV-2 infection affecting a significant portion of patients [6,7]. It may present with a wide range of physical or mental symptoms and complaints [17,18]. In our cohort, we found a significant improvement by 3 months and, given the paucity of follow-up due to cancelled appointments, we suspect that the majority of patients had complete resolution by 6 months. While reports around the incidence of myocardial involvement, including myocarditis, myocardial ischemia, or cardiomyopathy, are highly variable in the setting of COVID-19 disease [19,20], in our study, we were unable to demonstrate any significant structural or functional cardiac abnormality on echocardiography or with laboratory testing. However, this may be related to the fact that this was a study among outpatients who presented to us on average 4.6 ± 0.1 months after the acute infection, thus allowing enough time for functional and biochemical normalization.

As anticipated in the setting of a viral respiratory infection, DLCO values were reduced at the time of initial visit, which is similar to prior reports [21,22]. We were able to document a significant improvement in pulmonary function tests by the follow-up visit at 3 months, which is in line with the findings by Sonnweber and colleagues [23]. Confirming prior study data, DLCO and FVC values both showed a significant association with infection severity and longer hospital length of stay [24]. They also showed arterial oxygen desaturation on their 6MWT.

Another interesting finding in our study is related to lupus anticoagulant. This is a factor that causes thrombophilia and has an established role in autoimmune diseases. It is part of the three principal antiphospholipid antibodies with anti-cardiolipin and β2-glycoprotein [25]. We found that lupus anticoagulant levels were positive in about 25% of our population at the initial visit, which approaches the frequency observed in the population suffering from systemic lupus erythematosus (15–34%). The prevalence in the normal population is estimated to be at around 2–4% [26]. There is some evidence in the literature supporting that LA (and other antiphospholipid antibody) levels may be elevated during and following the acute phase of COVID-19 infection. Moreover, there is a direct proportionality between the severity of acute infection and the level of LA; however, a mild disease could also result in persistent LA positivity [27,28,29,30,31]. Though the role of antiphospholipid antibodies in hypercoagulability during COVID-19 infection is still under debate, in our study, we found a considerable, yet not significant tendency between thromboembolic complications and LA positivity [29,32]. However, the exact pathomechanism is still unclear; therefore, further studies are needed to define their role in the development of post-COVID syndrome.

Our work has several important limitations we need to mention. First, the referral process by general practitioners could have introduced a selection bias, including the likelihood of referral by the GPs and the willingness of patients to have a further examination by specialists. Asymptomatic patients and those with mild symptoms potentially did not seek medical attention, leading to underrepresentation of this population. Given, however, the overall number of infections in Hungary and Worldwide, healthcare systems would not have been able to accommodate all individuals with COVID-19. In addition, many could even be completely unaware of their infection. This is a likely limitation of all studies enrolling patients with SARS-CoV-2 infection. Second, a relatively low proportion of patients were admitted in critical condition, limiting the ability to draw meaningful conclusions about this cohort. Third, this is a single-center study in a large academic center with a population that may not be representative of the entire country. Fourth, only 12% of participants were current smokers, which is significantly lower than that reported in Hungary based on Eurostat data (26%) [33]. This may suggest that the patients presenting to our clinic were more health conscious and aware, therefore skewing the data. Fifth, despite our initial plans to follow all patients for 12 months, it was only possible for 3 months. This is due to the fact that post-COVID symptoms resolved for most individuals after 3 months, and therefore the majority did not return to the clinic to complete their follow-up visits. This limits the long-term follow-up of our study. Sixth, this particular study focused primarily on the cardiopulmonary characteristic of patients with post-COVID; therefore, potential neurological symptoms and deficits were not accurately evaluated.

## 5. Conclusions

The majority of patients presenting with post-COVID syndrome to our highly specialized, referral-only outpatient clinic were middle-aged, otherwise healthy individuals with low to moderate cardiovascular risk and mild prior COVID-19 infection. Those requiring hospitalization during the acute phase had compromised pulmonary function and more often persistent symptoms, such as dyspnea and fatigue.

Our study underlines some important and already-known facts about post-COVID syndrome; however, it also provides some important novelties for this research field. Hungary, a Central European country, was severely affected by the COVID-19 pandemic; in spite of this fact, we have only limited information regarding the Hungarian post-COVID population. Therefore, we aimed to increase this knowledge with this article. One of our interesting findings was the low rate of cardiovascular pathologies despite the extensive literature data about the common involvement of the cardiovascular system. Another interesting finding was a high rate of lupus anticoagulant positivity in this population, which can highlight the significance of autoimmune processes in the background of post-COVID syndrome. This could serve as a basis for further research in the future to better understand the underlying pathomechanisms. Our study also underlines the importance of inhaler treatments and pulmonary rehabilitation for affected individuals.

## Figures and Tables

**Table 1:** Comorbidities and their association with acute COVID-19 disease severity.

Comorbidity	Total	Treated at Home	Hospitalized	OR ^a^	*p*-Value ^a^
Diabetes mellitus	15.1%	11.4%	23.7%	2.42	**0.012**
Hypertension	42.8%	35.2%	60.5%	2.82	**<0.001**
COPD	1.2%	0.6%	2.6%	3.93	0.22
Bronchial asthma	8.7%	8.5%	9.2%	1.09	0.81
Thromboembolic events	5.9%	5.7%	6.6%	1.17	0.78

OR: Odds Ratio, ^a^ *p*-values and ORs were calculated using the Chi-squared test. Significant data are highlighted in bold.

**Table 2:** Most common post-COVID symptoms at the initial clinic visit.

Symptom/Complaint	Frequency
Fatigue, weakness, or reduced exercise tolerance	74 (29.4%)
Dyspnea	48 (19.1%)
Chest pain	27 (10.7%)
Dry cough	27 (10.7%)
Arrhythmias or palpitation	15 (5.9%)
Musculoskeletal symptoms	14 (5.6%)
Neurological symptoms	14 (5.6%)
High or unstable blood pressure	7 (2.8%)
Other (hair loss, urticaria, fever, gastro-intestinal symptoms)	9 (3.6%)

**Table 3:** Echocardiographic and pulmonary function test results.

	Baseline	Month 3	*p*-Value ^a^
LVEF (%)	62 (58–66)	64 (60–66)	0.28
LVIDd (mm)	45 (42–49)	44 (42–50)	0.48
LVIDs (mm)	27 (26–32)	28 (25–30)	0.43
Mitral e’	0.08 (0.07–0.11)	0.09 (0.07–0.12)	0.52
Mitral E/e’	7.88 (6.14–9.25)	7.64 (5.65–11.61)	0.53
FVC (% of expected)	101 (92–112)	99 (91–108)	0.37
FEV1 (% of expected)	99 (89–108)	96 (90–105)	0.12
Tiffeneau-index (% of expected)	81 (78–85)	81 (77–84)	0.19
DLCO (% of expected)	72 (64–86)	74 (66–86)	**0.028**

LVEF: Left ventricular ejection fraction; LVIDd: left ventricular internal dimension in diastole; LVIDs: left ventricular internal dimension in systole; *p*-values were calculated using Mann–Whitney-U test; FVC: forced vital capacity; FEV1: forced expiratory volume in the first second; DLCO: diffusion lung capacity. ^a^ *p*-values were calculated with Mann–Whitney-U test. Significant data are highlighted in bold.

**Table 4:** Changes in laboratory parameters during the 3-month follow-up period.

	Baseline Value	Abnormal (%)	3 Months	Abnormal (%)	*p*-Value ^a^
D-dimer (ug/L)	435 (310–719)	37.7	388 (274–593)	34.8	**<0.01**
Ferritin (ug/L)	113 (50–248)	18.1	106 (48–218)	10.9	**<0.01**
Fibrinogen (g/L)	3.4 (2.9–3.8)	24.2	3.3 (2.8–4.0)	25.6	0.96
hs-CRP (mg/L)	1.7 (1–3.1)	19.1	1.8 (0.8–4.4)	17.9	0.27
hs-Troponin-T (ng/L)	6.1 (3.5–9.0)	11.2	5.2 (3.7–7.6)	8.4	**<0.001**
IL-6 (pg/mL)	40 (10–320)	n.a.	73 (6–376)	n.a.	0.49
IL-33 (pg/mL)	200 (107–1064)	n.a.	279 (99–719)	n.a.	0.75
NT-proBNP (pg/mL)	70 (33–129)	5.0	72 (34–112)	4.4	0.36

hs: high sensitive; CRP: C-reactive protein; IL: interleukin; n.a.: not applicable; NT-proBNP: N-terminal pro-brain natriuretic peptide. ^a^ *p*-values were calculated between the results at baseline and 3 months using Mann–Whitney-U test. Significant data are highlighted in bold.

**Table 5:** Severity of acute COVID-19 infection between patient cohorts divided by the median age.

Severity Categories	Patients ≤ 52 Years	Patients > 52 Years
Treated at home	109 (82.6%)	67 (55.8%)
Hospitalized without respiratory failure	9 (6.8%)	4 (3.3%)
Hospitalized with respiratory failure	10 (7.6%)	40 (33.3%)
Intensive care admission	4 (3.0%)	9 (7.5%)

## Data Availability

Data are contained within the article or Supplementary Materials.

## Supplementary Materials

The following supporting information can be downloaded at: https://www.mdpi.com/article/10.3390/jcm14082604/s1, Table S1, Supporting information for Table 1; Tables S2 and S3, Supporting information for Table 3; Table S4, Supporting information for Figure 1; Table S5, Supporting information for Figure 2; Table S6, Supporting information for Table 4; Table S7, Supporting information for Figure 3; Table S8, Supporting information for Figure 4; Table S9, Supporting information for Table 5 and Figure 5.

## Abbreviations

The following abbreviations are used in this manuscript:

6MWT	6-Minute Walk Test
BMI	Body Mass Index
BNP	Brain Natriuretic Peptide
CAC	COVID-19-Associated Coagulopathy
COPD	Chronic Obstructive Pulmonary Disease
COVID	Coronavirus Disease
CRP	C-Reactive Protein
DLCO	Diffusion Lung Capacity for Carbon Monoxide
DM	Diabetes Mellitus
ECG	Electrocardiogram
ERS	European Respiratory Society
ESC	European Society of Cardiology
FEV1	Forced Expiratory Volume in the First Second
FVC	Forced Vital Capacity
GE	General Electric
GP	General Practitioner
hs	High Sensitivity
HT	Hypertension
ICU	Intensive Care Unit
Ig	Immunoglobulin
IL	Interleukin
IHME	Institute for Health Metrics and Evaluation
LA	Lupus Anticoagulant
LV	Left Ventricular
LVEF	Left Ventricular Ejection Fraction
LVIDd	Left Ventricular Internal Diameter in Diastole
LVIDs	Left Ventricular Internal Diameter in Systole
OR	Odds Ratio
NTproBNP	N-terminal proBNP
PACS	Post-Acute COVID-19 Syndrome
rt-PCR	Real time-Polymerase Chain Reaction
SARS-CoV-2	Severe Acute Respiratory Syndrome-Coronavirus-2
SEM	Standard Error of the Mean
TTE	Transthoracic Echocardiography
