# Supplementary material for: Prevalence and Cardiopulmonary Characteristics of Post-COVID Syndrome at a Hungarian Tertiary Referral Hospital
Source: J Clin Med. 2025 Apr 10;14(8):2604. doi: 10.3390/jcm14082604 (PMC12028108; doi:10.3390/jcm14082604)
Supplement: Supplementary file 1 [file jcm-14-02604-s001.zip › S1_Supporting information for Table 1.pdf]

Table S1. Supporting information for Table 1.

Participant	Hospitalized	Diabetes	Hypertension	COPD	Asthma	Thrombembolic events
1	No	No	No	No	No	No
2	No	No	Yes	No	No	No
3	Yes	No	Yes	No	No	No
4	Yes	No	Yes	No	No	No
5	Yes	Yes	Yes	No	No	No
6	No	No	No	No	No	No
7	No	No	No	No	No	No
8	No	No	No	No	No	No
9	No	No	Yes	No	No	No
10	Yes	No	Yes	No	No	No
11	Yes	Yes	No	No	No	No
12	No	No	No	No	No	No
13	Yes	Yes	Yes	No	No	No
14	Yes	No	Yes	No	No	No
15	Yes	No	Yes	No	No	No
16	No	No	No	No	Yes	No
17	No	No	No	No	No	No
18	No	No	Yes	No	No	No
19	Yes	No	Yes	No	No	No
20	No	No	No	No	No	No
21	No	No	Yes	No	No	No
22	Yes	No	No	Yes	Yes	No
23	No	No	No	No	No	No
24	No	No	Yes	No	No	No
25	No	No	No	No	No	No
26	Yes	No	Yes	No	No	No
27	No	No	No	No	No	No
28	Yes	No	No	No	No	No
29	Yes	No	No	No	No	No
30	No	No	No	No	No	No
31	Yes	No	Yes	No	No	No
32	No	No	No	No	No	Yes
33	No	No	No	No	No	No
34	No	No	No	No	No	No
35	No	No	No	No	No	No
36	No	No	No	No	No	Yes
37	No	No	No	No	No	No
38	No	No	No	No	No	No
39	Yes	Yes	No	No	No	No
40	No	No	No	No	No	No
41	Yes	No	No	No	No	No
42	No	No	Yes	No	No	No
43	No	Yes	Yes	No	No	No
44	Yes	Yes	Yes	No	No	No

45	Yes	No	No	No	No	No
46	No	No	No	No	No	No
47	No	No	No	No	No	No
48	No	No	No	No	No	Yes
49	No	No	No	No	No	No
50	Yes	No	No	No	No	No
51	No	No	No	No	Yes	No
52	No	No	No	No	No	No
53	No	No	No	No	No	No
54	No	No	No	No	No	No
55	No	No	No	Yes	Yes	No
56	Yes	No	Yes	Yes	Yes	No
57	No	No	No	No	No	No
58	Yes	No	Yes	No	No	No
59	Yes	Yes	Yes	No	No	Yes
60	Yes	No	No	No	No	No
61	No	No	No	No	No	Yes
62	No	Yes	Yes	No	No	No
63	Yes	No	Yes	No	No	No
64	Yes	No	Yes	No	No	No
65	No	No	No	No	No	No
66	Yes	No	No	No	No	No
67	No	Yes	Yes	Yes	Yes	No
68	No	Yes	No	No	No	No
69	Yes	No	No	No	No	No
70	No	No	Yes	No	No	No
71	No	No	Yes	No	No	No
72	No	Yes	Yes	No	No	No
73	No	No	Yes	No	No	No
74	No	No	No	Yes	Yes	No
75	No	No	Yes	No	No	No
76	No	No	No	No	No	No
77	Yes	No	Yes	Yes	No	No
78	Yes	No	Yes	No	No	No
79	No	No	No	No	No	No
80	No	No	Yes	No	No	No
81	No	No	No	No	No	No
82	No	No	No	No	No	No
83	No	No	No	No	No	No
84	No	No	No	No	No	No
85	No	No	Yes	No	No	No
86	No	No	No	No	No	No
87	No	No	Yes	No	No	No
88	No	Yes	Yes	No	No	No
89	No	No	No	No	No	No
90	No	No	No	No	No	No

91	No	No	No	No	No	No
92	No	No	No	No	No	No
93	Yes	No	No	No	No	No
94	Yes	No	Yes	No	No	No
95	No	No	No	No	No	No
96	Yes	No	Yes	No	No	No
97	No	No	Yes	No	No	No
98	Yes	Yes	Yes	No	No	No
99	No	No	No	No	No	No
100	No	No	No	No	Yes	No
101	No	No	No	No	No	No
102	No	No	No	No	No	No
103	No	No	No	No	No	No
104	No	No	No	No	No	No
105	No	No	Yes	No	No	No
106	Yes	No	Yes	No	No	No
107	No	No	No	No	No	No
108	No	No	Yes	Yes	Yes	No
109	No	No	No	No	No	No
110	No	Yes	Yes	No	No	No
111	No	Yes	Yes	No	No	No
112	No	No	No	No	No	No
113	Yes	No	Yes	No	No	No
114	No	No	No	No	No	No
115	No	No	No	No	No	No
116	No	No	No	No	No	No
117	No	No	Yes	No	No	Yes
118	Yes	Yes	Yes	No	No	No
119	No	No	No	No	No	No
120	No	Yes	Yes	No	No	No
121	No	No	Yes	No	No	No
122	No	No	No	No	No	No
123	No	No	Yes	No	No	No
124	Yes	No	Yes	No	No	No
125	Yes	No	Yes	No	No	No
126	Yes	No	No	Yes	Yes	No
127	Yes	Yes	Yes	No	No	No
128	Yes	Yes	No	No	No	No
129	Yes	No	No	Yes	Yes	No
130	No	No	No	No	No	No
131	No	No	No	No	No	No
132	Yes	No	No	No	No	Yes
133	No	No	Yes	No	No	No
134	No	Yes	Yes	No	No	No
135	Yes	No	No	No	No	Yes
136	No	No	Yes	No	No	No

137	Yes	No	Yes	No	No	No
138	No	No	No	No	No	No
139	Yes	Yes	Yes	No	No	No
140	No	No	No	No	No	Yes
141	No	Yes	Yes	No	No	No
142	Yes	No	No	No	No	No
143	Yes	No	Yes	No	No	No
144	No	No	No	No	No	No
145	No	No	No	No	No	No
146	No	No	No	No	No	No
147	Yes	No	Yes	No	No	No
148	No	No	Yes	No	No	No
149	No	No	No	No	No	No
150	No	No	No	No	No	No
151	No	No	Yes	No	No	No
152	No	No	No	Yes	Yes	No
153	Yes	Yes	No	No	No	No
154	No	Yes	Yes	No	No	No
155	Yes	No	Yes	No	No	No
156	No	No	No	No	No	No
157	Yes	No	Yes	Yes	No	No
158	No	No	No	No	No	No
159	No	No	No	No	No	No
160	Yes	No	No	No	No	No
161	No	No	No	No	No	No
162	No	No	Yes	No	No	No
163	Yes	Yes	Yes	No	No	No
164	No	No	Yes	No	No	No
165	No	No	No	No	No	No
166	Yes	No	Yes	Yes	Yes	No
167	No	Yes	Yes	No	No	No
168	No	No	Yes	No	Yes	No
169	Yes	No	Yes	No	No	No
170	No	No	No	No	No	No
171	No	No	No	No	No	No
172	Yes	No	Yes	Yes	Yes	No
173	No	No	No	No	No	No
174	Yes	No	Yes	Yes	Yes	No
175	No	Yes	No	No	No	Yes
176	Yes	Yes	Yes	No	No	No
177	No	No	Yes	No	Yes	No
178	No	No	Yes	No	No	No
179	Yes	Yes	No	No	No	No
180	No	No	No	No	No	No
181	Yes	No	No	No	No	No
182	Yes	No	No	No	No	No

183	No	Yes	No	No	No	No
184	No	No	No	Yes	No	No
185	No	No	No	Yes	Yes	No
186	No	No	Yes	No	No	No
187	No	No	Yes	No	No	No
188	No	No	Yes	No	No	No
189	No	No	Yes	No	No	No
190	No	No	Yes	No	No	No
191	Yes	No	Yes	No	No	Yes
192	No	No	Yes	No	No	No
193	No	No	No	No	No	No
194	Yes	Yes	Yes	No	No	No
195	Yes	Yes	Yes	No	No	No
196	No	No	Yes	No	No	No
197	No	No	No	No	No	No
198	No	No	No	No	No	No
199	No	No	Yes	No	No	No
200	No	No	Yes	No	No	No
201	Yes	No	No	No	No	No
202	No	No	No	No	No	No
203	No	No	No	No	No	No
204	No	No	No	No	No	No
205	No	Yes	Yes	No	Yes	No
206	No	No	Yes	No	No	No
207	Yes	Yes	Yes	No	No	No
208	No	No	No	No	No	No
209	No	No	No	No	Yes	No
210	No	No	No	No	No	No
211	No	No	No	No	No	No
212	No	No	No	No	No	No
213	No	Yes	Yes	No	No	No
214	No	No	No	No	No	No
215	No	No	No	No	No	No
216	No	No	Yes	No	No	No
217	Yes	No	No	No	No	No
218	No	No	No	No	No	No
219	No	No	No	No	No	No
220	Yes	No	No	No	No	Yes
221	Yes	No	No	No	No	No
222	No	No	Yes	No	No	No
223	No	No	No	No	No	No
224	No	No	No	No	No	No
225	No	No	Yes	No	No	No
226	No	No	No	No	No	No
227	No	No	No	Yes	Yes	No
228	No	Yes	Yes	No	No	No

229	No	No	No	No	No	No
230	Yes	No	No	No	No	No
231	Yes	No	Yes	No	No	No
232	Yes	No	Yes	No	No	No
233	No	No	No	No	No	No
234	No	No	No	No	No	No
235	Yes	No	No	No	No	No
236	No	No	No	No	No	No
237	No	Yes	No	No	No	No
238	No	No	No	No	No	No
239	No	No	Yes	No	No	No
240	No	No	No	No	No	No
241	No	No	Yes	No	No	No
242	No	No	No	No	No	No
243	No	No	Yes	No	Yes	No
244	No	Yes	Yes	No	No	No
245	No	No	Yes	No	No	Yes
246	Yes	No	No	No	No	No
247	No	No	Yes	No	No	Yes
248	No	No	No	No	No	No
249	No	No	No	No	No	No
250	No	No	No	No	No	No
251	Yes	No	Yes	No	No	No
252	No	No	No	No	No	No
