# Supplementary material for: Prevalence and Cardiopulmonary Characteristics of Post-COVID Syndrome at a Hungarian Tertiary Referral Hospital
Source: J Clin Med. 2025 Apr 10;14(8):2604. doi: 10.3390/jcm14082604 (PMC12028108; doi:10.3390/jcm14082604)
Supplement: Supplementary file 1 [file jcm-14-02604-s001.zip › S2_Supporting information for Table 3_ECHOCARDIOGRAPHY.pdf]

Table S2. Supporting information for Table 3.

ECHOCARDIOGRAPHY

Table S2A. Systolic parameters:

Participant	LVEF_Baseline (%)	LVEF_Month 3 (%)	LVIDd_Baseline (mm)	LVIDd_Month 3 (mm)	LVIDs_Baseline (mm)	LVIDs_Month 3 (mm)
1	69	70	36	44	20	24
2	66	66	38	41	22	24
3	80	64	41	50	18	30
4	71		41		22	
5	76	63	43	41	21	25
6	68		48		27	
7	61		40		25	
8	56	72	50	47	33	25
9	64		50		30	
10	58	64	48	50	31	30
11	62	61	55	48	34	30
12	58		48		31	
13	65	65	44	49	26	29
14	62	57	42	46	26	30
15	56		51		34	
16	72	60	38	36	20	23
17	57	69	46	45	26	25
18	63		47		32	
19	57	54	52	44	34	30
20	62	63	42	38	26	23
21	63		48		30	
22	64	64	45	50	27	30
23	64		52		31	
24	68		51		29	
25	62	56	47	48	29	32
26	60		46		29	
27	61	60	45	44	28	28
28	58		43		28	
29	43	52	61	58	46	40
30	64		45		27	
31	65		44		26	
32	62	59	42	47	26	30
33	68		46		26	
34	60	60	41	40	26	26
35	69		41		23	
36	66		41		24	
37	59	60	39	38	25	24
38	72		38		20	
39	65		44		26	

40	72		53		28	
41	72	62	45	44	24	27
42	69		38		19	
43	62	60	44	44	27	28
44	68		44		25	
45	61		51		32	
46	61		37		23	
47	60		41		26	
48	66		48		28	
49	71		39		21	
50	71		43		23	
51	62		39		24	
52	62	57	42	44	26	29
53	61		48		30	
54	68		44		25	
55	72		38		20	
56	69	75	43	48	24	25
57	64		45		27	
58	55		49		33	
59	63		41		25	
60	66		38		22	
61	60		46		29	
62	56	56	48	50	32	33
63	59	66	42	44	27	26
64	61	63	51	48	32	29
65	68		44		25	
66	65		42		25	
67	62	63	47	43	29	26
68	65		39		23	
69	60		49		31	
70	67		40		23	
71	61		45		28	
72	65		42		25	
73	66	64	46	45	27	27
74	66	64	55	50	32	30
75	61		52		33	
76	61	67	43	42	27	24
77	52	66	36	43	25	25
78	60		52		33	
79	66	73	41	42	24	22
80	71	63	41	41	22	25
81	60		46		29	
82	62		47		29	
83	75	68	40	37	20	21
84	25		67		60	
85	54	58	40	43	27	28

86	66		43		25	
87	66		41		24	
88	67		51		32	
89	58		43		28	
90	69		43		24	
91	59		47		30	
92	60		45		28	
93	64		40		24	
94	65	67	47	42	28	24
95	61	58	43	37	27	24
96	63	57	47	49	31	32
97	61		43		27	
98	51	68	50	44	35	25
99	63		46		28	
100	60		52		33	
101	71		52		28	
102	68	52	34	42	19	29
103	58		43		28	
104	62		47		29	
105	64		45		27	
106	69		50		28	
107	62		52		32	
108	64	65	40	42	24	25
109	74	58	39	43	20	28
110	58	63	43	48	30	29
111	57	51	49	53	32	37
112	73		46		24	
113	63		48		29	
114	56	64	47	50	31	30
115	48		50		36	
116	54	61	40	42	27	25
117	66	69	43	36	25	20
118	56	40	57	60	38	41
119	61		40		25	
120	61		40		28	
121	59		42		27	
122	63		43		26	
123	59	66	47	41	30	24
124	61	65	43	42	27	25
125	60		49		31	
126	54	64	53	50	36	30
127	64		40		24	
128	68		39		22	
129	65		54		32	
130						
131	58		45		29	

132	61		48		30		
133	67		49		28		
134	68		53		30		
135				45			
136	70		41		21		
137	60		45		29		
138	62		44		27		
139	59		54		34		
140	63		48		29		
141	54		53		36		
142	61		46		28		
143	50	64	41	50	29	30	
144	64	72	45	38	27	20	
145	68	67	37	35	21	20	
146	73		44		23		
147	62	61	55	51	34	32	
148	69		41		23		
149	58		54		35		
150	64		45		27		
151	61	58	53	54	33	35	
152	58		40		26		
153	69	69	41	45	23	25	
154	53		54		37		
155	54	66	50	38	34	22	
156	76		41		20		
157	68		44		25		
158	67		40		23		
159	65		49		29		
160	60	76	46	41	29	19	
161							
162	70	65	49	47	27	28	
163	70		49		27		
164	67		47		27		
165	63		41		25		
166	56		54		36		
167	59	67	50	45	32	26	
168	60		46		29		
169	63		43		26		
170	61	54	48	44	30	30	
171	53	64	41	40	28	24	
172	53	64	51	40	35	24	
173	61	65	48	49	30	29	
174	62		47		29		
175	58		54		35		
176	60	63	44	38	28	23	
177							

178	56		56		37	
179	67		42		24	
180	67		42		24	
181	62	69	47	41	29	23
182	64		46		30	
183	55	57	55	52	35	34
184						
185	72		40		21	
186	67	66	40	50	23	29
187	62	69	44	50	27	28
188	70	63	49	46	27	28
189	63		43		26	
190	53		35		24	
191	68	68	39	44	22	25
192	63	66	51	46	31	27
193	74		41		21	
194	58		45		29	
195	53	49	48	53	33	38
196	57	61	49	40	32	25
197	63	60	46	44	28	28
198	57		49		32	
199	62		40		24	
200	56	50	51	55	34	39
201	62	67	44	42	27	27
202	72		45		24	
203	57		46		30	
204	61		48		30	
205	59	51	47	50	30	35
206	64	64	45	50	27	30
207	54		53		36	
208	61	66	45	46	28	27
209	67		40		23	
210	66		43		25	
211	66		38		22	
212	64		50		30	
213	65	66	47	43	28	25
214	63		56		34	
215	70		49		29	
216	66		43		25	
217	66	64	49	50	27	30
218	60		42		27	
219	61		40		25	
220	62	68	42	39	26	22
221	55	64	50	50	34	30
222	63	59	56	56	34	36

223	62		47		29	
224	63	61	46	48	28	30
225	56		53		35	
226	63		49		30	
227	65	61	44	48	26	30
228	59		56		36	
229	61		48		30	
230	56		51		34	
231	50	47	50	55	33	40
232	73		60		31	
233	69		47		26	
234	61		48		30	
235	64		45		27	
236	63		41		25	
237	61	64	40	40	25	24
238						
239	51	62	50	44	35	27
240	65		44		26	
241	63		49		30	
242	65		37		22	
243	69		45		25	
244	69	64	45	40	25	24
245	65		49		29	
246	61	61	43	45	27	28
247	67	58	56	48	32	31
248	70		44		26	
249	63		41		25	
250	63	63	46	46	28	28
251	67	66	35	36	20	21
252						

Table S2B. Diastolic parameters:

Participant	e'_Baseline (ms)	e'_Month 3 (ms)	E/e'_Baseline	E/e'_Month 3
1	0,08	0,08	8,13	7,50
2	0,08	0,06	10,00	1,00
3	0,11		5,45	
4	0,08		10,00	
5	0,09	0,07	10,00	8,57
6				
7	0,14		5,71	
8	0,16	0,12	5,94	5,83
9				
10	0,08		6,63	

11	0,13		6,15	
12				
13	0,06	0,06	13,33	16,67
14	0,1	0,1	8,60	5,00
15	0,06		10,00	
16	0,12		7,92	
17	0,13	0,13	6,15	6,92
18	0,14		6,00	
19	0,1		5,10	
20		0,14		8,89
21	0,08		10,00	
22	0,05	0,05	13,40	13,00
23	0,14		5,29	
24				
25				
26	0,08		10,00	
27		0,12		7,50
28	0,04		20,00	
29	0,07	0,09	5,71	5,56
30	0,17		4,71	
31	0,05		22,00	
32	0,14	0,2	6,43	4,50
33	0,09		10,00	
34	0,9		0,98	
35	0,12		5,42	
36	0,1		7,50	
37	0,11	0,17	8,00	0,47
38	0,14		5,71	
39				
40	0,13		7,69	
41	0,07	0,09	17,43	3,33
42	0,09		6,67	
43	0,06		8,33	
44				
45				
46	0,1		6,50	
47	0,08		10,00	
48				
49	0,12		7,50	
50	0,09		7,56	
51	0,08		10,63	
52		0,12		8,33
53	0,14		5,00	
54	0,16		5,63	
55	0,13		7,92	
56	0,09	0,08	5,56	7,50
57	0,12		4,17	
58	0,07		12,14	
59				
60	0,05		9,00	

61	0,09		7,78	
62	0,07	0,12	1,63	11,67
63	0,09	0,06	5,44	12,50
64	0,08	0,06	8,38	8,33
65	0,07		8,57	
66	0,07		7,14	
67	0,08	0,06	8,13	11,67
68	0,15		3,33	
69				
70	0,08		7,50	
71	0,18		5,00	
72	0,06		10,00	
73	0,07	0,1	5,71	8,00
74	0,11	0,06	5,91	15,83
75	0,09		10,00	
76		0,13		9,23
77	0,08	0,1	11,25	9,00
78	0,08		10,00	
79	0,12	0,11	6,50	7,27
80	0,08	0,09	10,63	8,89
81	0,14		6,43	
82				
83	0,16	0,13	5,63	6,15
84				
85	0,07	0,06	5,71	10,00
86	0,07		10,00	
87	0,08		11,25	
88	0,09		10,11	
89	0,17		7,24	
90	0,11		8,18	
91	0,1		6,50	
92				
93	0,08		8,75	
94	0,06	0,08	8,33	8,75
95	0,09	0,09	5,56	7,22
96	0,06	0,06	8,67	12,50
97	0,06		5,83	
98	0,1	0,05	8,00	12,00
99				
100	0,19		3,68	
101	0,15		5,00	
102	0,08		12,50	
103	0,12		5,83	
104				
105				
106	0,07		8,57	
107				
108	0,11	0,06	8,36	13,33
109	0,14		7,86	
110	0,05	0,07	16,00	5,71

111	0,05	0,05	18,00	22,00
112	0,09		6,11	
113	0,08		6,25	
114	0,11	0,1	7,36	8,00
115	0,04		15,00	
116	0,15	0,2	6,13	4,25
117	0,14	0,16	6,21	5,63
118	0,08	0,12	7,50	5,00
119	0,16		6,25	
120				
121	0,11		7,45	
122	0,15		5,67	
123	0,11		6,36	
124	0,05		10,80	
125	0,05		6,00	
126	0,12		6,67	
127				
128	0,13		4,62	
129				
130				
131	0,13		6,15	
132				
133	0,1		6,50	
134	0,13		4,62	
135		0,07		8,57
136	0,07		8,57	
137				
138				
139	0,09		5,56	
140				
141	0,04		10,00	
142	0,09		8,89	
143	0,07	0,11	7,14	5,45
144		0,14		5,00
145		0,11		8,18
146	0,06		6,67	
147				
148	0,05		14,00	
149	0,16		6,25	
150	0,16		4,06	
151	0,08	0,12	11,25	6,67
152				
153	0,12	0,14	6,67	5,71
154	0,08		6,25	
155	0,08	0,08	5,63	7,50
156	0,15		5,00	
157	0,05		9,00	
158	0,11		8,18	
159				
160		0,06		10,00

161				
162	0,11	0,12	8,18	5,42
163	0,08		12,50	
164	0,07		14,29	
165				
166	0,08		10,38	
167		0,05		6,00
168				
169	0,07		10,00	
170	0,6	0,1		9,00
171	0,08		8,75	
172	0,11	0,07	7,27	7,14
173	0,11	0,15	7,64	4,67
174				
175				
176	0,05	0,07	12,00	71,43
177				
178				
179	0,05		13,00	
180	0,15		5,67	
181		0,12		6,67
182	0,05		8,00	
183	0,09	0,08	8,56	13,75
184				
185	0,12		5,00	
186	0,09	0,13	9,44	6,92
187	0,08	0,13	7,50	6,15
188	0,08		6,88	
189	0,05		14,00	
190	0,08		10,00	
191	0,09		10,56	
192	0,08	0,05	10,00	10,00
193	0,11		7,27	
194	0,08		10,00	
195	0,08	0,07	7,13	10,00
196	0,11	0,1	8,64	10,00
197	0,21	0,19	5,24	4,74
198	0,07		5,71	
199	0,09		8,89	
200	0,06	0,06	8,33	11,67
201	0,15	0,12	5,33	5,42
202				
203	0,09		6,44	
204	0,09		8,89	
205	0,06	0,09	8,33	7,78
206	0,08	0,05	8,25	14,00
207	0,08		7,50	
208		0,17		4,12
209	0,11		4,55	
210	0,13		6,15	

211	0,15		7,33	
212				
213	0,07	0,07	10,00	8,57
214	0,13		4,62	
215	0,08		13,50	
216	0,08		6,25	
217	0,08	0,08	7,50	6,25
218				
219	0,13		7,31	
220	0,09	0,1	5,56	8,00
221	0,08		8,75	
222	0,08		6,25	
223				
224				
225	0,06		10,00	
226				
227	0,11		6,36	
228	0,08		8,13	
229	0,18		4,44	
230	0,12		5,83	
231	0,05	0,08	15,00	12,50
232				
233	0,13		5,38	
234				
235	0,16		5,50	
236	0,12		6,67	
237	0,08	0,15	7,75	4,67
238				
239	0,12	0,07	6,83	11,43
240				
241				
242	0,12		9,00	
243	0,07		9,29	
244				
245	0,06		10,00	
246	0,1		7,40	
247	0,13		3,85	
248	0,1		5,00	
249	0,13		5,38	
250	0,12		4,17	
251	0,05		12,00	
252				
