# Supplementary material for: Prevalence and Cardiopulmonary Characteristics of Post-COVID Syndrome at a Hungarian Tertiary Referral Hospital
Source: J Clin Med. 2025 Apr 10;14(8):2604. doi: 10.3390/jcm14082604 (PMC12028108; doi:10.3390/jcm14082604)
Supplement: Supplementary file 1 [file jcm-14-02604-s001.zip › S3_Supporting information for Table 3_PULM.pdf]

Table S3. Supporting information for Table 3.

PULMONARY FUNCTION TESTING

Participant	FVC_Baseline (%)	FVC_Month 3 (%)	FEV1_Baseline (%)	FEV1_Month 3 (%)	TI_Baseline (%)	TI_Month 3 (%)	DLCO_Baseline (%)	Dlco_Month 3 (%)
1	90,2	88,8	83,4	82,1	79,45	79,54	63,9	65,9
2	108,1	104,4	103,6	103,2	79,53	81,84	58,8	64
3	96,5	86,2	88,3	80,1	70,91	72	64,2	58,6
4	91,2		95		83,28		70,2	
5	107,2	107,7	107,8	106,8	85,28	84,02	67	73,5
6	78,4		77,2		85,1		72,1	
7	113,6		108,8		80,85		86,7	
8	104,3	103,6	100,9	101,3	83,82	84,65	84,3	91,6
9	82,6		85,5		82,17		70,9	
10	98,9	89,2	99	92	81,49	83,99	93,9	93,1
11	97,4	96,7	95,3	95	77,43	77,53	69,6	82,7
12	112,8		103,8		80,39		81	
13	95,4	95,4	95,4	103,2	78,54	84,89	64,7	65,5
14	93,1	97,2	91,4	96,8	81,3	82,5	52,2	56,4
15	84,6		90,7		83,98		56,4	
16	93,6	99,5	91,9	95,9	84,78	83,23	55,7	59,7
17	87	87,5	81,9	83,1	76,25	77,06	72	80
18	113,6		114,6		82,62		90,7	
19	83,7	55,9	71,7	54,8	65,63	75,01	28,2	33,3
20	115,4	106,2	108,1	99,1	79,93	79,44	70,1	63,2
21								
22	75,8	92,5	76,3	96,9	78,78	81,95	67,8	82
23	83,2		88		87,4		68,9	
24	87,4		87,7		81,14		76,4	
25	121,2	123,5	111,8	114,4	78,14	78,42	97,8	105,5
26	99,7		94,7		81,78		88,2	

27	100	100,4	90,1	89,1	77,95	76,85	71,6	71,2
28	105,9		96,6		77,65		71,5	
29	97,5	105,7	104,1	109,5	81,47	79	79,4	86,6
30	106,9		94,7		76,18		85,2	
31	100,6		93,3		71,83		52,5	
32	87,1		75,2		74,06		52,5	
33	99,3		99,8		83,18		103,5	
34	100,4	107,7	101,9	105,4	81,85	78,71	85,3	85,4
35								
36	142,8		144,3		82,71		95,2	
37	128	128	116	116	77,95	77,95	74	74
38	67,4		68,2		86,15			
39	91,8		96,7		89,25		65,2	
40	96,1		102,3		88,1		74	
41	98,1	93	99	93,6	78,16	77,72	67,2	60
42	101		39,6		74,35		70,7	
43	101,9	96,4	106,4	99,9	88	87,18	75,4	72,6
44	117		121		86,21		90	
45	104,9		108,7		80,04		87,9	
46	117,1		119,4		85,18		81,7	
47	132,9		122		77,14		84,4	
48	107,4		101,3		81		76,1	
49	111,1		111,6		86,86		88,6	
50	110,3		107,6		83,94		84,9	
51	104,1		94,5		78,55		65,6	
52	98,8	95,5	102,3	102,1	89,38	92,26	78,5	78,6
53	106,2		95,3		77,32		67,1	
54	92,3		91,9		87,1		66,6	
55	113		110		84,32		74	
56	78,2	91,7	66	85,7	61,57	67,96	74,7	72,1

57	118,5		120,5		85,08		72,9	
58	114,3		102,2		73,29		86	
59	98,4		88,1		73,98		87,1	
60	118		121,9		84,74		76,2	
61	103,7		92		75,57		61,7	
62	142	93,7	145	100,4	84,9	89,41	73	85,7
63	131,1	136,6	134,2	140,2	83,94	84,17	104,2	73,7
64	99	98,7	109	104,5	88,04	84,1	69	77,4
65	94,5		104,2		85,23		83,2	
66	89,6		86,6		76,46		75,7	
67	88,6	87,4	83,8	83,6	80,46	81,42	68,9	69,1
68	89		83,5		81,33		54,3	
69	54,2		56,8		85,09		43,8	
70	89,3		94,7		91,23		70,1	
71	96,2		106,3		86,95		93,7	
72	104,8		97,5		79,29		84,6	
73	95,6	98,2	88	91	78,96	79,51	89,9	87,1
74	111	109,9	96	93,9	73,3	72,3	86	86,3
75	117,8		108		79,67		102,9	
76	95,6	101	92,9	94,4	83,41	80,22	69,8	64,4
77	112	108,9	84	75,6	63,09	58,4	53,2	50,1
78	95		92,2		75,13		60,5	
79	113	109,8	118	104,8	88,41	81,15	82	80,6
80	112,6	117,9	110,2	107,4	81,95	76,28	75,2	77,5
81	118,9		111,4		76,62		77,5	
82	103,1		98,1		78,73		92	
83	109,8	113,6	111,9	115,4	89,1	88,71	85,7	74,5
84	88		77,7		71,19		87,1	
85	133,9	141,6	134	133,6	82,27	77,16	64,1	60
86	106,2		97		78,42		77,6	

87	95,5		85,1		76,1		61,7	
88								
89	117		97		72,56		81	
90	102,5		97,8		82,88		73,3	
91	98		94,7		79,86		106,7	
92	92,5		98		88,63		90,4	
93	94,3		101,1		85,92		65	
94	101,5	106,2	95,9	102,4	77,66	79,16	80,2	85,7
95	116	111,5	108,4	108,8	79,4	82,77	82,4	90,6
96	86,1	88,7	93,4	95,2	86,06	85,21	45,8	70,3
97	103,5		104,1		77,72		96,4	
98	90,3	98,6	102,8	108,9	87,61	84,67	77,5	75,5
99	94,6		93,1		81,29		86,3	
100	100,3		98		81,15		85	
101	130,2		109,7		73,84		83	
102	107	118,3	109,1	110,3	86,96	79,52	75,9	71,5
103	118,5		111,5		81,5		73,5	
104	83,1		70,5		70,32		44,8	
105	107,7		106,4		73,86		78,2	
106	72,9		78,2		92,25		77,5	
107	99,4		100,4		82,83		80,5	
108	111,4	100,9	98,8	90,9	74,41	75,66	5,67	84,6
109	88,4	120,1	85,2	90,2	83,23	64,84	87	91,3
110		84,4		88		79,3		107
111	71,9	81,1	67,8	73,1	78,79	75,23	48,3	41,1
112	84,7		89,6		86,54		70,6	
113	87,5		87,5		85,93		64,4	
114	104,4	97,4	99,5	95,7	77,71	80,14	83,1	70,4
115	85		79,1		78,4		66,8	
116	103,2	87,5	89	90,6	74,92	89,96	89,7	101,6

117	100,5	93,7	94,1	88,2	80,72	81,2	70,9	74,1
118	73,2	76,8	74,4	74,9	79,13	75,96	53,7	52,7
119	89,6		84,4		81,78		79,4	
120	98,7		89,6		78,12		83,8	
121	113		112		82,2		68	
122	124,9		118,1		79,56		94,3	
123	116,4	117,2	110,9	114,3	81,4	83,39	87,3	89,1
124	87,9	89,5	93,2	94	87,93	86,99	38,3	36
125	97,4		102,9		79,78		75,3	
126	101,5	101,2	105,7	103,9	85,44	84,29	86	104,1
127	116,7		111,1		77,35		66,2	
128	71,9		81,6		90,95		39,9	
129	86,4		92,8		86,13		63,5	
130	92,5		103,1		97,37		70,5	
131	100,9		103,5		84,47		83,1	
132	89,6		85,3		81,36		73,6	
133	86,2		81,6		79,11		60,7	
134	95,4		90,6		82,01		83,2	
135	115,2	107,8	105,4	95,6	77,2	74,79	85,7	89,1
136	99,3		100,4		86,09		104,5	
137	102,1		101,4		79,42		82,7	
138	111,7		102,7		71,37		75,3	
139	88,3		78,2		70,02		73,5	
140	97,7		100,8		83,69		65,3	
141	85,1		100,6		90,04		88,4	
142	126		122		82,05		83	
143	34,2	64,8	38,7	72,8	88,3	87,53	33,5	50
144	114,2	106,9	102,3	94,8	77,88	77,12	68,9	71,2
145	97,3		89,3		79,01		88,2	
146	133,2		120,8		74,8		74,5	

147	98	93,5	100	98,2	82,79	85,07	71	70,4
148	115,9		109,6		79,36		86,9	
149	114		109,6		79,91		97,7	
150	96,1		97,3		82,01		109,3	
151	84	85,3	72,9	73,8	68,56	68,33	100,4	101,7
152	114		107,1		81,1		55,6	
153	108,3	105,2	105,4	97,7	84,52	80,68	60,7	66,8
154	116,6		106,4		73,63		94,5	
155	100,5	102,4	95,7	96,4	81,66	80,78	84,3	75,4
156	95		91,8		83,81		69,2	
157	93,3		67,6		56,9		31,7	
158	116,4		117,9		86,62		65,7	
159	112,8		114,1		87,64		76,6	
160	120,9	120,9	138,7	138,7	89,46	89,46	69,3	69,3
161	92,6		84,1		78,39		67,7	
162	93,3	88,4	93,3	84,4	79,84	76,25	93,9	87,7
163	108,3		108,2		84,55		76,1	
164	105,6		106,2		86,85		92,1	
165	100,8		94		80,7		74,5	
166	90,5		82,4		78,4		75,4	
167	122,2	116,2	114,7	108,9	78,48	78,24	92,4	91,9
168	107,3		87,6		70,18		83,5	
169	92,3		92,6		84,61		56,3	
170	83,8	92,6	81,4	88,9	78,61	77,5	64,6	66,3
171	92,7	98,8	92,4	93,2	84,54	80,12	72,4	70,2
172	84,9	94,9	79,6	97	74,21	80,89	58,4	71
173	123	107,5	112	112	78,66	90,5	82	79,2
174	88		93,2		82,01		76,8	
175	110,8		106,8		74,55		73,2	
176	107,3	103,9	100,8	94,6	79,08	76,69	92,1	85,6

177	90,6		93,4		89,22		77,3	
178	137,5		124,3		77,06		101,7	
179	86,5		100,6		83,43		66,6	
180	81,3		88,3		93,28		91,5	
181	103	125,8	107	121,6	83,42	77,79	64	74,1
182	77,7		85,5		84,68		78,7	
183	114,4	101,2	103,2	86,7	76,96	73,21	6,17	66,2
184	75,3		66,7		75,09		60,8	
185	118		97,4		70,78		96,1	
186	98	92,7	100	90,1	86,96	82,88	57,4	55,5
187	99,3	94,3	105,7	101,8	86,11	87,2	102,2	101,9
188	65,1	74,9	72,2	75,7	90,28	82,34	60,7	63,3
189	102,8		97		79,13		80,1	
190	88,2		56,5		53,48		63,3	
191	93,5	111,7	90,2	104,4	81,37	78,85	70,1	71
192	130	127,4	127,2	123	81,94	80,85	79,3	78,5
193	91,2		83,9		79,02		73,8	
194	93,8		103,3		86,26		96,5	
195	67	69,9	70	70,2	82,69	79,78	54	56
196	107,9	96,9	113,8	102	83,34	83,2	101	106,9
197	128	120,1	122	114,3	82,35	82,61	89	83
198	99,9		95,5		75,27		82	
199	141,1		128,2		77,45		91,1	
200	87,8	87,9	96,9	91,3	86,36	81,04	71,7	67,3
201	120	113,4	114	106,9	81,36	80,9	66	62,9
202	108,9		107,9		83,7		68,1	
203	105,9		114,4		85,99		81,5	
204	88,3		82		76,75		88,6	
205	100,4	103,2	90,7	92,3	77,07	76,32	101,5	95
206	69,3	65,5	62	58,9	72,14	72,57	61,9	73,8

207	74,1		74,5		78,75		60,1	
208	100,6	94,5	95,4	94,9	82,3	87,03	83,6	85,5
209	114,8		102,4		74,5		88,2	
210	98,9		102		87,64		75,4	
211	87,6		86,9		85,96		72,8	
212	121		114,7		75,33		86,4	
213	113,3	111,3	93	90,4	66	65,3	93	94,4
214	110,2		110,5		85,73		59,6	
215	106,2		92,2		74,63		73,2	
216	93,4		95,6		87,16		70,7	
217	95,2	90,3	103,3	97,3	86,42	85,87	85,3	85,2
218	119,3		109,4		78,68		94,7	
219	110		106		84,28		61	
220	81,4	89,9	89,3	96,5	90,97	88,98	60,1	73,4
221	56	75,6	64	85,1	90,1	88,82	38	56,5
222	77,8	53,9	84,7	63,2	87,33	93,91	79,6	69,4
223	107,1		105,3		82,87		95,9	
224		102,5		106,9		87,07		78,5
225	114,5		112		83,06		96,4	
226	100,8		95,5		78,29		77,6	
227	105,5	98,3	93,2	88,5	75,81	76,96	67,6	71,2
228	60,5		64,4		85,03		66,4	
229	77,5		78,8		85,62		94,1	
230	84		91,2		88,46		100,8	
231	101,8	100,5	99,5	98,4	81,78	81,98	63,4	65,9
232	65,8		70		87,06		87	
233	94,1		91,2		81,02		92,2	
234	101,8		102,9		88,38		74,5	
235	101,9		99,1		82,45		94,6	
236	122,8		111,5		78,2		85,2	

237	117	107,8	109	97,7	79,27	77,45	76	80
238	141,6		131,8		77,92		109,7	
239	106,1	93,7	108,7	95,6	80,11	79,77	90,1	90,1
240	113,3		125,1		96		62,4	
241	86		81,9		79,95		50,3	
242	92,2		83,3		76,61		70	
243	76,9		65		71		85,8	
244	135,3	136,1	137,3	139,1	82,13	82,74	71,8	76,7
245	110,7		136,3		94,36		61,2	
246	100,8	96,8	105,4	102,7	82,58	83,82	83,9	94,7
247	106,9	106,9	98,2	101,3	73,05	75,4	102,9	100,1
248	127,5		125,8		84,07		85,1	
249	119,6		111,6		80,5		81	
250	95,4	71,8	88,6	79,9	72,45	86,47	94	92,4
251	95,4		108,4		88,93		40,9	
252	90,4		86,9		83,56		65,2	
