# Supplementary material for: Prevalence and Cardiopulmonary Characteristics of Post-COVID Syndrome at a Hungarian Tertiary Referral Hospital
Source: J Clin Med. 2025 Apr 10;14(8):2604. doi: 10.3390/jcm14082604 (PMC12028108; doi:10.3390/jcm14082604)
Supplement: Supplementary file 1 [file jcm-14-02604-s001.zip › S4_Supporting information for Figure 1.pdf]

Figure S4. Supporting information for Figure 1.

Participant	Infection severity	FVC (%)	Dlco (%)
1	Treated at home	90,2	63,9
2	Treated at home	108,1	58,8
3	Hospitalized with respiratory failure	96,5	64,2
4	Hospitalized with respiratory failure	91,2	70,2
5	Hospitalized with respiratory failure	107,2	67
6	Treated at home	78,4	72,1
7	Treated at home	113,6	86,7
8	Treated at home	104,3	84,3
9	Treated at home	82,6	70,9
10	Hospitalized with respiratory failure	98,9	93,9
11	Hospitalized with respiratory failure	97,4	69,6
12	Treated at home	112,8	81
13	Hospitalized with respiratory failure	95,4	64,7
14	Hospitalized with respiratory failure	93,1	52,2
15	Hospitalized with respiratory failure	84,6	56,4
16	Treated at home	93,6	55,7
17	Treated at home	87	72
18	Treated at home	113,6	90,7
19	Required intensive care	83,7	28,2
20	Treated at home	115,4	70,1
21	Treated at home		
22	Hospitalized with respiratory failure	75,8	67,8
23	Treated at home	83,2	68,9
24	Treated at home	87,4	76,4
25	Treated at home	121,2	97,8
26	Hospitalized with respiratory failure	99,7	88,2
27	Treated at home	100	71,6

28	Hospitalized without respiratory failure	105,9	71,5
29	Hospitalized without respiratory failure	97,5	79,4
30	Treated at home	106,9	85,2
31	Hospitalized with respiratory failure	100,6	52,5
32	Treated at home	87,1	52,5
33	Treated at home	99,3	103,5
34	Treated at home	100,4	85,3
35	Treated at home		
36	Treated at home	142,8	95,2
37	Treated at home	128	74
38	Treated at home	67,4	
39	Hospitalized with respiratory failure	91,8	65,2
40	Treated at home	96,1	74
41	Hospitalized with respiratory failure	98,1	67,2
42	Treated at home	101	70,7
43	Treated at home	101,9	75,4
44	Hospitalized with respiratory failure	117	90
45	Required intensive care	104,9	87,9
46	Treated at home	117,1	81,7
47	Treated at home	132,9	84,4
48	Treated at home	107,4	76,1
49	Treated at home	111,1	88,6
50	Hospitalized without respiratory failure	110,3	84,9
51	Treated at home	104,1	65,6
52	Treated at home	98,8	78,5
53	Treated at home	106,2	67,1
54	Treated at home	92,3	66,6
55	Treated at home	113	74
56	Hospitalized without respiratory failure	78,2	74,7
57	Treated at home	118,5	72,9

58	Hospitalized without respiratory failure	114,3	86
59	Hospitalized with respiratory failure	98,4	87,1
60	Hospitalized with respiratory failure	118	76,2
61	Treated at home	103,7	61,7
62	Treated at home	142	73
63	Hospitalized with respiratory failure	131,1	104,2
64	Hospitalized with respiratory failure	99	69
65	Treated at home	94,5	83,2
66	Hospitalized with respiratory failure	89,6	75,7
67	Treated at home	88,6	68,9
68	Treated at home	89	54,3
69	Hospitalized with respiratory failure	54,2	43,8
70	Treated at home	89,3	70,1
71	Treated at home	96,2	93,7
72	Treated at home	104,8	84,6
73	Treated at home	95,6	89,9
74	Treated at home	111	86
75	Treated at home	117,8	102,9
76	Treated at home	95,6	69,8
77	Hospitalized without respiratory failure	112	53,2
78	Hospitalized with respiratory failure	95	60,5
79	Treated at home	113	82
80	Treated at home	112,6	75,2
81	Treated at home	118,9	77,5
82	Treated at home	103,1	92
83	Treated at home	109,8	85,7
84	Treated at home	88	87,1
85	Treated at home	133,9	64,1
86	Treated at home	106,2	77,6
87	Treated at home	95,5	61,7

88	Treated at home		
89	Treated at home	117	81
90	Treated at home	102,5	73,3
91	Treated at home	98	106,7
92	Treated at home	92,5	90,4
93	Required intensive care	94,3	65
94	Hospitalized with respiratory failure	101,5	80,2
95	Treated at home	116	82,4
96	Required intensive care	86,1	45,8
97	Treated at home	103,5	96,4
98	Hospitalized with respiratory failure	90,3	77,5
99	Treated at home	94,6	86,3
100	Treated at home	100,3	85
101	Treated at home	130,2	83
102	Treated at home	107	75,9
103	Treated at home	118,5	73,5
104	Treated at home	83,1	44,8
105	Treated at home	107,7	78,2
106	Hospitalized with respiratory failure	72,9	77,5
107	Treated at home	99,4	80,5
108	Treated at home	111,4	5,67
109	Treated at home	88,4	87
110	Treated at home		
111	Treated at home	71,9	48,3
112	Treated at home	84,7	70,6
113	Required intensive care	87,5	64,4
114	Treated at home	104,4	83,1
115	Treated at home	85	66,8
116	Treated at home	103,2	89,7
117	Treated at home	100,5	70,9

118	Hospitalized with respiratory failure	73,2	53,7
119	Treated at home	89,6	79,4
120	Treated at home	98,7	83,8
121	Treated at home	113	68
122	Treated at home	124,9	94,3
123	Treated at home	116,4	87,3
124	Hospitalized with respiratory failure	87,9	38,3
125	Required intensive care	97,4	75,3
126	Hospitalized with respiratory failure	101,5	86
127	Hospitalized with respiratory failure	116,7	66,2
128	Hospitalized with respiratory failure	71,9	39,9
129	Hospitalized with respiratory failure	86,4	63,5
130	Treated at home	92,5	70,5
131	Treated at home	100,9	83,1
132	Hospitalized without respiratory failure	89,6	73,6
133	Treated at home	86,2	60,7
134	Treated at home	95,4	83,2
135	Hospitalized with respiratory failure	115,2	85,7
136	Treated at home	99,3	104,5
137	Hospitalized with respiratory failure	102,1	82,7
138	Treated at home	111,7	75,3
139	Hospitalized with respiratory failure	88,3	73,5
140	Treated at home	97,7	65,3
141	Treated at home	85,1	88,4
142	Hospitalized without respiratory failure	126	83
143	Required intensive care	34,2	33,5
144	Treated at home	114,2	68,9
145	Treated at home	97,3	88,2
146	Treated at home	133,2	74,5
147	Hospitalized with respiratory failure	98	71

148	Treated at home	115,9	86,9
149	Treated at home	114	97,7
150	Treated at home	96,1	109,3
151	Treated at home	84	100,4
152	Treated at home	114	55,6
153	Hospitalized without respiratory failure	108,3	60,7
154	Treated at home	116,6	94,5
155	Hospitalized with respiratory failure	100,5	84,3
156	Treated at home	95	69,2
157	Hospitalized with respiratory failure	93,3	31,7
158	Treated at home	116,4	65,7
159	Treated at home	112,8	76,6
160	Hospitalized with respiratory failure	120,9	69,3
161	Treated at home	92,6	67,7
162	Treated at home	93,3	93,9
163	Hospitalized with respiratory failure	108,3	76,1
164	Treated at home	105,6	92,1
165	Treated at home	100,8	74,5
166	Hospitalized without respiratory failure	90,5	75,4
167	Treated at home	122,2	92,4
168	Treated at home	107,3	83,5
169	Hospitalized with respiratory failure	92,3	56,3
170	Treated at home	83,8	64,6
171	Treated at home	92,7	72,4
172	Required intensive care	84,9	58,4
173	Treated at home	123	82
174	Hospitalized with respiratory failure	88	76,8
175	Treated at home	110,8	73,2
176	Hospitalized with respiratory failure	107,3	92,1
177	Treated at home	90,6	77,3

178	Treated at home	137,5	101,7
179	Required intensive care	86,5	66,6
180	Treated at home	81,3	91,5
181	Hospitalized with respiratory failure	103	64
182	Hospitalized with respiratory failure	77,7	78,7
183	Treated at home	114,4	6,17
184	Treated at home	75,3	60,8
185	Treated at home	118	96,1
186	Treated at home	98	57,4
187	Treated at home	99,3	102,2
188	Treated at home	65,1	60,7
189	Treated at home	102,8	80,1
190	Treated at home	88,2	63,3
191	Hospitalized with respiratory failure	93,5	70,1
192	Treated at home	130	79,3
193	Treated at home	91,2	73,8
194	Hospitalized with respiratory failure	93,8	96,5
195	Hospitalized with respiratory failure	67	54
196	Treated at home	107,9	101
197	Treated at home	128	89
198	Treated at home	99,9	82
199	Treated at home	141,1	91,1
200	Treated at home	87,8	71,7
201	Hospitalized without respiratory failure	120	66
202	Treated at home	108,9	68,1
203	Treated at home	105,9	81,5
204	Treated at home	88,3	88,6
205	Treated at home	100,4	101,5
206	Treated at home	69,3	61,9
207	Required intensive care	74,1	60,1

208	Treated at home	100,6	83,6
209	Treated at home	114,8	88,2
210	Treated at home	98,9	75,4
211	Treated at home	87,6	72,8
212	Treated at home	121	86,4
213	Treated at home	113,3	93
214	Treated at home	110,2	59,6
215	Treated at home	106,2	73,2
216	Treated at home	93,4	70,7
217	Hospitalized without respiratory failure	95,2	85,3
218	Treated at home	119,3	94,7
219	Treated at home	110	61
220	Hospitalized with respiratory failure	81,4	60,1
221	Required intensive care	56	38
222	Treated at home	77,8	79,6
223	Treated at home	107,1	95,9
224	Treated at home		
225	Treated at home	114,5	96,4
226	Treated at home	100,8	77,6
227	Treated at home	105,5	67,6
228	Treated at home	60,5	66,4
229	Treated at home	77,5	94,1
230	Hospitalized without respiratory failure	84	100,8
231	Hospitalized with respiratory failure	101,8	63,4
232	Required intensive care	65,8	87
233	Treated at home	94,1	92,2
234	Treated at home	101,8	74,5
235	Required intensive care	101,9	94,6
236	Treated at home	122,8	85,2
237	Treated at home	117	76

238	Treated at home	141,6	109,7
239	Treated at home	106,1	90,1
240	Treated at home	113,3	62,4
241	Treated at home	86	50,3
242	Treated at home	92,2	70
243	Treated at home	76,9	85,8
244	Treated at home	135,3	71,8
245	Treated at home	110,7	61,2
246	Hospitalized with respiratory failure	100,8	83,9
247	Treated at home	106,9	102,9
248	Treated at home	127,5	85,1
249	Treated at home	119,6	81
250	Treated at home	95,4	94
251	Hospitalized with respiratory failure	95,4	40,9
252	Treated at home	90,4	65,2
