# Supplementary material for: Prevalence and Cardiopulmonary Characteristics of Post-COVID Syndrome at a Hungarian Tertiary Referral Hospital
Source: J Clin Med. 2025 Apr 10;14(8):2604. doi: 10.3390/jcm14082604 (PMC12028108; doi:10.3390/jcm14082604)
Supplement: Supplementary file 1 [file jcm-14-02604-s001.zip › S5_Supporting information for Figure 2.pdf]

Figure S5. Supporting information for Figure 2.

Participant	Desaturation	FVC (%)	Dlco (%)
1	No	90,2	63,9
2	No	108,1	58,8
3	No	96,5	64,2
4	No	91,2	70,2
5	No	107,2	67
6	No	78,4	72,1
7	No	113,6	86,7
8	No	104,3	84,3
9	No	82,6	70,9
10	No	98,9	93,9
11	No	97,4	69,6
12	No	112,8	81
13	No	95,4	64,7
14	No	93,1	52,2
15	Yes	84,6	56,4
16	No	93,6	55,7
17	No	87	72
18	No	113,6	90,7
19	No	83,7	28,2
20	No	115,4	70,1
21			
22	Yes	75,8	67,8
23	No	83,2	68,9
24	No	87,4	76,4
25	No	121,2	97,8
26	No	99,7	88,2
27	No	100	71,6
28	No	105,9	71,5
29	No	97,5	79,4
30	Yes	106,9	85,2
31	No	100,6	52,5
32		87,1	52,5
33	No	99,3	103,5
34	No	100,4	85,3
35	No		
36	No	142,8	95,2
37	No	128	74
38	Yes	67,4	
39	No	91,8	65,2
40	No	96,1	74
41	No	98,1	67,2
42	Yes	101	70,7
43	No	101,9	75,4
44	No	117	90

45		104,9	87,9
46	Yes	117,1	81,7
47	No	132,9	84,4
48	No	107,4	76,1
49	No	111,1	88,6
50	No	110,3	84,9
51	No	104,1	65,6
52	No	98,8	78,5
53	No	106,2	67,1
54	No	92,3	66,6
55	No	113	74
56	Yes	78,2	74,7
57	No	118,5	72,9
58		114,3	86
59		98,4	87,1
60	No	118	76,2
61	No	103,7	61,7
62	No	142	73
63	Yes	131,1	104,2
64	No	99	69
65	No	94,5	83,2
66	Yes	89,6	75,7
67	No	88,6	68,9
68	No	89	54,3
69	Yes	54,2	43,8
70	No	89,3	70,1
71	No	96,2	93,7
72	No	104,8	84,6
73	No	95,6	89,9
74	No	111	86
75	No	117,8	102,9
76	No	95,6	69,8
77	No	112	53,2
78	No	95	60,5
79	No	113	82
80	No	112,6	75,2
81	No	118,9	77,5
82	No	103,1	92
83	No	109,8	85,7
84	No	88	87,1
85	No	133,9	64,1
86	No	106,2	77,6
87	No	95,5	61,7
88	No		
89	No	117	81
90	No	102,5	73,3

91		98	106,7
92	No	92,5	90,4
93	No	94,3	65
94	No	101,5	80,2
95		116	82,4
96	No	86,1	45,8
97	No	103,5	96,4
98	No	90,3	77,5
99	No	94,6	86,3
100	No	100,3	85
101	No	130,2	83
102	No	107	75,9
103	No	118,5	73,5
104	No	83,1	44,8
105	No	107,7	78,2
106	No	72,9	77,5
107	No	99,4	80,5
108	No	111,4	5,67
109	No	88,4	87
110	No		
111	No	71,9	48,3
112	No	84,7	70,6
113	No	87,5	64,4
114	No	104,4	83,1
115	Yes	85	66,8
116	No	103,2	89,7
117	Yes	100,5	70,9
118	Yes	73,2	53,7
119	No	89,6	79,4
120	No	98,7	83,8
121	No	113	68
122	No	124,9	94,3
123	No	116,4	87,3
124	Yes	87,9	38,3
125	No	97,4	75,3
126	No	101,5	86
127	No	116,7	66,2
128	No	71,9	39,9
129		86,4	63,5
130	Yes	92,5	70,5
131	No	100,9	83,1
132	No	89,6	73,6
133	No	86,2	60,7
134	No	95,4	83,2
135	No	115,2	85,7
136	No	99,3	104,5

137	No	102,1	82,7
138	No	111,7	75,3
139	No	88,3	73,5
140	No	97,7	65,3
141		85,1	88,4
142	No	126	83
143	No	34,2	33,5
144	No	114,2	68,9
145	No	97,3	88,2
146	No	133,2	74,5
147	No	98	71
148	No	115,9	86,9
149	No	114	97,7
150	No	96,1	109,3
151	No	84	100,4
152	No	114	55,6
153	No	108,3	60,7
154	No	116,6	94,5
155	No	100,5	84,3
156	No	95	69,2
157		93,3	31,7
158	No	116,4	65,7
159	Yes	112,8	76,6
160	No	120,9	69,3
161	Yes	92,6	67,7
162	No	93,3	93,9
163	No	108,3	76,1
164	No	105,6	92,1
165	No	100,8	74,5
166	No	90,5	75,4
167	No	122,2	92,4
168	No	107,3	83,5
169	No	92,3	56,3
170	No	83,8	64,6
171	No	92,7	72,4
172	Yes	84,9	58,4
173	No	123	82
174	No	88	76,8
175	No	110,8	73,2
176	No	107,3	92,1
177	No	90,6	77,3
178	Yes	137,5	101,7
179	No	86,5	66,6
180	No	81,3	91,5
181	No	103	64
182	No	77,7	78,7

183	No	114,4	6,17
184	No	75,3	60,8
185	No	118	96,1
186	No	98	57,4
187	No	99,3	102,2
188	No	65,1	60,7
189	No	102,8	80,1
190	No	88,2	63,3
191		93,5	70,1
192	No	130	79,3
193	No	91,2	73,8
194	No	93,8	96,5
195	Yes	67	54
196	No	107,9	101
197	No	128	89
198	No	99,9	82
199	No	141,1	91,1
200	No	87,8	71,7
201	No	120	66
202	No	108,9	68,1
203	No	105,9	81,5
204		88,3	88,6
205	No	100,4	101,5
206	No	69,3	61,9
207	Yes	74,1	60,1
208	No	100,6	83,6
209	No	114,8	88,2
210	No	98,9	75,4
211	No	87,6	72,8
212	No	121	86,4
213	No	113,3	93
214	No	110,2	59,6
215	No	106,2	73,2
216	No	93,4	70,7
217	No	95,2	85,3
218	No	119,3	94,7
219	No	110	61
220	No	81,4	60,1
221	Yes	56	38
222	No	77,8	79,6
223	No	107,1	95,9
224	No		
225	No	114,5	96,4
226	No	100,8	77,6
227	Yes	105,5	67,6
228	No	60,5	66,4

229	No	77,5	94,1
230	No	84	100,8
231	No	101,8	63,4
232		65,8	87
233	No	94,1	92,2
234	No	101,8	74,5
235	No	101,9	94,6
236	No	122,8	85,2
237	Yes	117	76
238	No	141,6	109,7
239	No	106,1	90,1
240	No	113,3	62,4
241		86	50,3
242	No	92,2	70
243	No	76,9	85,8
244	No	135,3	71,8
245	Yes	110,7	61,2
246	No	100,8	83,9
247	No	106,9	102,9
248	No	127,5	85,1
249	No	119,6	81
250	No	95,4	94
251	No	95,4	40,9
252	Yes	90,4	65,2
