# Supplementary material for: Prevalence and Cardiopulmonary Characteristics of Post-COVID Syndrome at a Hungarian Tertiary Referral Hospital
Source: J Clin Med. 2025 Apr 10;14(8):2604. doi: 10.3390/jcm14082604 (PMC12028108; doi:10.3390/jcm14082604)
Supplement: Supplementary file 1 [file jcm-14-02604-s001.zip › S6_Supporting information for Table 4.pdf]

Table S6. Supporting information for Table 4.

Table S6A. Significantly changed:

Participant	D-dimer_Baseline	D-dimer_Month 3	Ferritin_Baseline	Ferritin_Month 3	Troponin_Baseline	Troponin_Month 3
1	262	47	113	65	3	6,92
2	2546		14	25	16,81	
3	408	335	297	304	4,94	3
4	250		285		6,95	
5	2553	542	193	73	7,84	6,93
6	311				8,18	
7	976		113		4,7	
8	239	203	115	106	5,42	3,96
9	954		214		11,6	
10	318	304	247	188	5	3,73
11	366	357	254	234	6,76	4,68
12	341		62		3	
13	469	493	89	96	32,91	40,64
14	774	491	306	281	7,72	4,63
15	394		599		10,2	
16	497	304	98	83	3	3
17	755	858	462	322	3	7,57
18	279		207		3	
19	3069	2848	58	44	11,32	7,55
20	263	754	71	54	5,9	4,97
21	288		174		7,18	
22	414	355	114	132	12,02	9,31
23	269		142		4,27	
24	513		290		6,97	
25	30	83	167	138	3,36	3

26	559		72		5,89	
27	507	474	21	38	6,25	4,55
28	158		92		3	
29	571	594	573	526	16,05	12,22
30	657		12		3,68	
31	1460		30		4,59	
32	476		66	11	3,33	5,96
33	418		217		3	
34	451	356	196	205	6,16	4,68
35	263				6,86	
36	235		159		5,99	
37	390	379	6	6	4,2	3
38	910		22		6,99	
39	360		45		16,71	
40	209		167		3,57	
41	763	604	177	218	7,93	7,06
42	532		358		5,55	
43	814	736	116	168	6,81	5,85
44	792		215		7,96	
45	111		104		7	
46	319		68		7,44	
47	1529		254		6,66	
48	641		171		3,29	
49	144		88		3	
50	535		15		3,6	
51	280		19		3	
52	383	326	32	18	3	3
53	1251		74		3,68	
54	621		56		5,08	

55	350		80		3	
56	956	420	198	318	34,75	34,52
57	1188		204		30,07	
58	298		118		9,91	
59	970		66		19,64	
60	1072		117		4,39	
61	353		7		4,32	
62	191	305	70	61	3,22	6,18
63	489	731	312	134	15,46	9,61
64	406	1034	232	86	10,07	6,69
65	206		655		3	
66	429		285		5,8	
67	906	488	112	117	5,36	7,58
68	325		32		3	
69	469		180		10,28	
70	545		79		3	
71	501		164		6,58	
72	325		247		5,68	
73	374	464	110	154	5,09	3,3
74	263	391	215	115	8,58	7,92
75	212		92		3,54	
76	544	309	33	18	4,02	3
77	261	165	123	128	7,63	5,48
78	365		115		6,88	
79	341	387	49	41	3	3
80	646	484	52	46	3	4,58
81	159		162		6,93	
82	552		74		3,31	
83	311	234	21	13	3	3
84	369		224		3,43	

85	463	142	164	94	9,63	7,71
86	375		11		5,86	
87	450		82		3	
88	278		511		7,3	
89	374		67		3,69	
90	1371		44		3	
91	257		143		3	
92	103		157		4,48	
93	338		564		14,56	
94	186	272	259	147	3	6,53
95	368	357	33	30	4,38	3
96	356	213	344	84	24,6	4,98
97	753		234		6,62	
98	330		544		16,72	
99	986		235		5,71	
100	149		123		11	
101	370		29		3	
102	563	544	39	64	3,92	3
103	310		65		4,05	
104	1186		151		13,7	
105	446				7,66	
106	442		107		4,93	
107	115				10,71	
108	547	508	78	97	3	4,14
109	236	236	30	17	4,83	7,02
110	1635	15675	155	117	12,22	10,5
111	1203		96	117	21,8	16,79
112	385		9		6,84	
113	251		52		4,24	
114	314	433	350	312	3	4,07

115	1419		308		5,46	
116	254	279	34	29	3	3,71
117	449	456	4	6	7,01	3,86
118	445	220	20	18	21,27	19,9
119	429		239		3	
120	156		57		3	
121	149		83		7,26	
122	195		60		3	
123	666	741	8	12	3,92	3
124	361	388		1096	9	19,05
125	1011		512		16,13	
126	381	314	307	177	15,6	6,97
127	511		185		4,3	
128	546		599		13,43	
129	1155				7,53	
130	315		43		3	
131	79		214		3	
132	193		87		3	
133	632		82		3	
134	520		89		3	
135	914	1351	50	48	7,02	5,69
136	419				3,35	
137	260		82		5,67	
138	247		121		11	
139	346		362		11,46	
140	386				3	
141	2487	794			25,3	
142	154		45		3,63	
143	1165	620	472	126	13,34	7,93
144	567	285	44	76	4,2	3

145	396	305	132	120	4,66	4,21
146	355		74		11,06	
147	186	280	40	66	5,84	4,43
148	378		127		3	
149	84		71		5,61	
150	400		380		3	
151	269	257	177	188	5,92	4,1
152	190				3	
153	325	53	17	11	3	6,32
154	2080		114		16,23	
155	788	678	248	255	4,17	3
156	623		204		4,68	
157	1594		254		3,45	
158	313		52		5,36	
159	405		63		6,55	
160	381	258	81	122	11,57	8,29
161	636		10		3	
162	748	481	217	235	7,77	7,69
163	781		239		4,13	
164	291		47		6,49	
165	402		34		3,23	
166	527		22		7,4	
167	737	569	191	191	6,14	3,82
168	740				3	
169	335		18		3	
170	425	207	66	48	17,37	17,95
171	609	970	98	89	5,96	4,71
172	887	766	220	232	8,89	9,84
173	1217		13	27	3	7,72
174	958		110		11,7	

175	274		74		7,26	
176	264	312	329	386	7,28	5,27
177	217		57		3	
178	1540		741		11,5	
179	396		255		14,34	
180	729		114		3,4	
181	132	176	579	267	11,34	4,35
182	491		240		17,92	
183	749	590	67	46	11,92	8,32
184	360		8		4,59	
185	178		16		5,8	
186	213	308	70	83	6,55	5,45
187	319	81	174	169	4,09	3,5
188	663		454	356	3,57	
189	349		116		4,6	
190	1488		131		7,2	
191	409	288	75	83	7,16	5,49
192	165	235	604	526	3,32	3,22
193	350		152		3,67	
194	431		300		27,25	
195	7567		723		21,04	
196	1087	983	495	357	7,19	8,7
197	422	484	64	49	3	3
198	446		475		11,86	
199	284		128		9,46	
200	549	535	228	317	6,95	4,76
201	486	355	7	40	4,13	3
202	217		87		10,24	
203	367		794		5,55	
204	278		162		3,28	

205	608	773	92	83	6,88	5,12
206	841	340	1689	1193	12,97	5,88
207	490	416	45		17,14	14,94
208	234	570	95	87	3,46	3
209	310		66		4,9	
210	98		234		5,35	
211	290		52		3	
212	314				3,09	
213	309	222	488	437	3	4,4
214	372		399		6,65	
215	402		29			
216	546		14		3	
217	425		830	654	3	3
218	155		46		3,52	
219	333		16		4,53	
220	5020	931	194	162	3,6	3
221	307	244	614	374	13,1	5,89
222	595	509	509	599	5,36	3,94
223	354		218		3,8	
224	273	243	83	82	3	3
225	570		296		6,02	
226	979		312		4,78	
227	484	233	39	32	3,76	3
228	775		506		4,13	
229	114		168		3,45	
230	284		120		4,92	
231	985	852	116	106	3	6,88
232	1147		52		15,94	
233	166		96		22,77	
234	298				8,05	

235	204		55		4,58	
236	351		22		4,24	
237	868	603	34	23	3	4,33
238	684		153		13,34	
239	302	557	642	511	14,13	11,82
240	401		71		4,84	
241	843				3,05	
242	295		86		5,4	
243	743				3,9	
244	570		26		8,26	
245	1009		24		176,4	
246	311	350	104	82	6,37	5,24
247	119	176	264	234	6,33	6,04
248	594		247		3	
249	701		62		3	
250	574	631	103,5	202	8,84	7,49
251	1844	1331	49	73	35,64	36,86
252	115	180	10	11	6,42	7,57

Table S6B. Did not change significantly:

Participant	CRP_Baseline	CRP_Month 3	Fibrinogen_Baseline	Fibrinogen_Month 3	NT-proBNP_Baseline	NT-proBNP_Month 3
1	2,4	2,3	2,73	2,73	167	146
2	0,6		2,76		78	
3	0,4	1,4	2,9	3,01	89	223
4	1,3		3,47		90	
5	4,4	5,6	3,32	3,76	62	71
6	6,8		4,91		27	
7	2,7		3,53		50	

8	4,7	7	3,05	2,86	27	53
9	6,8		5,02		41	
10	2,6	4,1	3,7	4,71	59	61
11	1,6	2,1	2,52	2,37	64	28
12	6,4		3,89		19	
13	32,91	2,6		4,26	162	227
14	3,5	5,8	3,96	4,18	180	166
15	1,6		3,83		122	
16	2,9	1,3	3,09	3,09	61	10
17	2,7	5	3,7	3,89	34	14
18	0,6		2,12		10	
19	1,8	0,6	4,52	3,64	262	94
20	1,8	1,7	3,09	3,23	45	86
21	3,2		2,86		488	
22	0,5	0,9	3,37	3,96	39	66
23	5,3		3,96		22	
24	4,8		4,52		10	
25	0,5	1,3	2,69	3,01	10	10
26	7		4,71		181	
27	1,4	7,9	3,27	3,42	86	105
28	1,5		2,9		95	
29	1,8	1,5	4,52	4,71	136	96
30	1,6		3,47		143	
31	4,3		4,81		131	
32	0,2	0,2	2,6	2,97	33	96
33	0,6		3,01		31	
34	3,3	5,3	3,7	3,64	18	16
35	1				78	
36	3,9		4,43		24	
37	0,2	0,3	2,93	2,52	33	75

38	2,2		3,59		30	
39	0,7		3,37		200	
40	0,7		2,3		10	
41	1,4	0,8	2,66	2,63	63	50
42	2,7		4,26		280	
43	3,2	5,3	3,76	4,52	77	105
44	3		3,7		597	
45	0,8		2,86		51	
46	0,8		3,96		151	
47	52,8		7,71		16	
48	4,4		3,89		120	
49	0,2		2		39	
50	0,9		3,96		30	
51	1,6		2,83		85	
52	0,2	0,7	2,32	2,39	78	74
53	7,9		3,58		45	
54	3,1		3,09		99	
55	4,8		4,03		16	
56	0,8	7,2	4,71	5,65	197	213
57	5,7		5,51		102	
58	4,4		3,37		247	
59	7,8		3,23		370	
60	1,3		4,18		31	
61	6,6		3,59		161	
62	2,1	1,9	3,14	2,86	123	1161
63	2,3	0,5	4,03	3,83	294	432
64	2,8	4,4	3,83	2,9	348	72
65	0,6		2,16		46	
66	1,2		3,7		19	
67	5	4,4	3,76	4,26	33	67

68	9,3		3,14		48	
69	3,7		4,35		23	
70	11,7		5,02		20	
71	1,3		2,55		18	
72	1,2		2,6		41	
73	8,4	10,2	3,83	4,26	58	18
74	4,3	3,2	3,18	3,37	142	112
75	2,2		4,03		42	
76	4,9	4,5	3,89	4,11	40	110
77	0,8	0,8	3,58	3,23	85	94
78	3,2		3,01		114	
79	3,1	5,5	3,76	3,76	47	40
80	1,1	1,2	3,7	3,37	119	131
81	0,3		2,35		32	
82	1,1		2,6		27	
83	1,9	0,9	2,76	1,89	139	83
84	3,7		3,01		536	
85	1,1	2,1		3,37	56	99
86	2,2		3,58		128	
87	3,3		3,32		10	
88	3,4		3,27		10	
89	0,8		2,26		36	
90	0,7		2,58		41	
91	1,5		3,83		10	
92	1,1		4,03		21	
93	2,2		3,58		75	
94	0,7	0,9	3,47	3,64	10	10
95	1,7	7,5	3,05	4,11	28	28
96	3,7	1,6	5,94	2,47	111	112
97	1		2,9		47	

98	3,1		3,32		80	
99	0,3		2,49		45	
100	2,1		3,12		17	
101	1,6		2,83		73	
102	0,3	0,2	3,27	3,18	144	122
103	0,2		2,24		40	
104	3,5		4,91		443	
105	0,8		2,35		125	
106	2,3		3,89		64	
107	4,2		3,14		43	
108	1,6	2,3	3,47	3,47	24	19
109	0,4	0,3	2,26	2,02	121	110
110	1,3	1,6	2,86	2,79	54	78
111	5,5	7,7			2003	2842
112	2,1		2,86		10	
113	6		3,96		141	
114	1,9	2,1	2,3	3,09	23	63
115	6,7		308		550	
116	2	1,9	2,93	2,49	76	45
117	1,5	0,8	3,58	3,27	38	45
118	1,9	1,3	2,73	2,49	254	121
119	1		4,11		275	
120	16,1		3,42		21	
121	0,3		2,69		390	
122	0,2		1,59		30	
123	1,2	0,5	3,76	3,01	98	29
124	1,9	6	3,53	4,03	152	381
125	3,7		6,82		182	
126	7,6	4	6,1	4,26	75	16
127	4,7		4,11		43	

128	5,7		3,96		74	
129	1		4,18		240	
130	1,6		3,01		38	
131	0,6		2,66		42	
132	5,4		5,02		45	
133	3,2		4,61		186	
134	5,8		3,53		29	
135	0,9	0,7	3,27	3,27	371	216
136	3,8				58	
137	0,9		2,44		94	
138	1,6		3,09		122	
139	4,2		3,76		35	
140	2		2,97		14	
141	12,2		4,71		97	
142	0,2		2,39		28	
143	5,2	3,9	7,02	5,14	129	135
144	0,7	0,4	2,86	2,73	79	99
145	1,1	0,4	2,93	2,58	322	411
146	1,2		4,09		212	
147	1,5	1,6	2,66	2,66	30	17
148	0,3		3,7		82	
149	0,3		2,32		10	
150	2,8		3,64		25	
151	1,4	1,8	3,01	3,58	53	49
152	0,5		4,03		77	
153	1,5	0,8	3,37	2,63	10	17
154	3,5		5,94		305	
155	4,7	4,4		5,14	30	90
156	4,7		3,58		69	
157	0,7		4,91		108	

158	5,8		4,61		116	
159	1,1		2,42		214	
160	0,5	0,8	2,73	2,73	29	16
161	0,7		2,9		194	
162	1,3	4,7	3,42	3,58	76	31
163	3,9		3,53		86	
164	6,6		5,26		29	
165	1,7		4,43		52	
166	9,6		3,89		45	
167	3,6	0,8	3,27	2,6	38	58
168	10,2		3,27		68	
169	4,4		3,37		35	
170	1,9	1,8	4,81	4,91	99	131
171	18	11,5	3,37	4,81	61	63
172	1,2	4	3,7	4,11	123	98
173	2,6	2,7	5,94		47	76
174	5,8		4,11		275	
175	56,7		4,11		58	
176	6,5	10,9	3,42	3,89	81	18
177	15,3		5,94		13	
178	8,7		4,61		41	
179	6,6		5,02		885	
180	0,7		3,83		32	
181	1,9	4,3	2,35	2,42	27	29
182	1,5		3,32		71	
183	24	22,7	4,03	4,35	154	182
184	1,4		2,28		113	
185	0,2		2,22		31	
186	1	1,3	3,09	2,97	98	117
187	0,4	0,2	2,86	2,9	21	15

188	15,4		5,14	4,61	24	
189	2,2		3,05		131	
190	1		3,47			
191	1,6	1,1	3,53	2,97	233	244
192	2,8	2	3,83	3,32	39	59
193	8,3		2,9		10	
194	2,5		4,11		121	
195	11,2		3,64		201	
196	1,1	1,2	2,93	3,23	29	31
197	2,6	2,8	3,53	3,27	120	84
198	1,5		3,47		152	
199	1,1		2,93		64	
200	0,5	0,8	2,42	3,47	285	108
201	0,6	0,8	2,6	2,6	19	37
202	4,5		2,42		202	
203	2,7		2,73		16	
204	2,8				18	
205	7,6	9	3,89	3,83	44	71
206	0,5	2,5	2,2	2,44	41	106
207	0,6	0,7	4,35	3,64	70	60
208	4,7	2,4	2,69	2,93	98	34
209	1,5		3,53		98	
210	0,9		2,76		18	
211	9,2		4,61		51	
212	0,6		3,89		37	
213	2,3	1,1	3,83	2,86	31	26
214	1,1		2,6		142	
215	0,2		2,32		92	
216	3,5		4,52		118	
217	1,5	0,7	2,97	2,52	20	45

218	0,4		1,79		79	
219	0,4		3,37		35	
220	2,2	1,4	3,32	2,83	175	168
221	1,5	1,9	3,89	4,43	198	60
222	1,4	3,4	3,89	5,02	27	65
223	0,6		2,55		24	
224	0,6	0,8	2,49	2,26	32	58
225	14,9		3,18		130	
226	17,1		4,91		116	
227	0,9	0,9	2,83	2,97	96	51
228	2,3		5,26		84	
229	0,5		2,03		39	
230	1,9		3,64		10	
231	30,4	4,6	5,02	3,53	469	970
232	11,7		5,02		94	
233	1,6		3,05		63	
234	7,8		4,61		35	
235	3,1		3,76		29	
236	1,4		2,2		30	
237	2,6	2,8	3,01	2,66	14	33
238	4,9				78	
239	1,2	1,9	3,76	3,76	70	132
240	0,6		2,83		95	
241	5,9		4,03		72	
242	0,5		3,52		210	
243	7,2				176	
244	0,9		4,11		175	
245	0,5		3,58		251	
246	0,6	1	3,64	4,43	10	11
247	0,6	0,5	3,37	4,03	28	31

248	2,8		2,9		41	
249	3,4		3,76		18	
250	1,7	0,7	4,11	3,64	72	103
251	4	5,2	3,18	4,52	765	322
252	1	0,3	2,96	3	62	67
