# Supplementary material for: Prevalence and Cardiopulmonary Characteristics of Post-COVID Syndrome at a Hungarian Tertiary Referral Hospital
Source: J Clin Med. 2025 Apr 10;14(8):2604. doi: 10.3390/jcm14082604 (PMC12028108; doi:10.3390/jcm14082604)
Supplement: Supplementary file 1 [file jcm-14-02604-s001.zip › S7_Supporting information for Figure 3.pdf]

Figure S7. Supporting information for Figure 3.

Participant	Length of hospital stay (days)	FVC (%)	DLCO (%)	6MWT - end saturation (%)
1	0	90,2	63,9	92
2	0	108,1	58,8	97
3	22	96,5	64,2	99
4	7	91,2	70,2	95
5	9	107,2	67	99
6	0	78,4	72,1	91
7	0	113,6	86,7	99
8	0	104,3	84,3	94
9	0	82,6	70,9	95
10	8	98,9	93,9	96
11	12	97,4	69,6	96
12	0	112,8	81	97
13	6	95,4	64,7	95
14	7	93,1	52,2	92
15	16	84,6	56,4	88
16	0	93,6	55,7	97
17	0	87	72	98
18	0	113,6	90,7	96
19	8	83,7	28,2	93
20	0	115,4	70,1	97
21	0			
22	0	75,8	67,8	86
23	0	83,2	68,9	98
24	0	87,4	76,4	95
25	0	121,2	97,8	99
26	7	99,7	88,2	97
27	0	100	71,6	94

28	7	105,9	71,5	98
29	19	97,5	79,4	93
30	0	106,9	85,2	85
31	0	100,6	52,5	91
32	0	87,1	52,5	
33	0	99,3	103,5	96
34	0	100,4	85,3	96
35	0			94
36	0	142,8	95,2	93
37	0	128	74	98
38	0	67,4		82
39	12	91,8	65,2	92
40	0	96,1	74	95
41	13	98,1	67,2	96
42	0	101	70,7	89
43	0	101,9	75,4	97
44	15	117	90	95
45	11	104,9	87,9	
46	0	117,1	81,7	86
47	0	132,9	84,4	96
48	0	107,4	76,1	97
49	0	111,1	88,6	99
50	1	110,3	84,9	99
51	0	104,1	65,6	93
52	0	98,8	78,5	99
53	0	106,2	67,1	98
54	0	92,3	66,6	95
55	0	113	74	97
56	10	78,2	74,7	80
57	0	118,5	72,9	96

58	5	114,3	86	
59	7	98,4	87,1	
60	8	118	76,2	93
61	0	103,7	61,7	99
62	0	142	73	95
63	17	131,1	104,2	87
64	6	99	69	96
65	0	94,5	83,2	96
66	9	89,6	75,7	89
67	0	88,6	68,9	97
68	0	89	54,3	94
69	15	54,2	43,8	77
70	0	89,3	70,1	97
71	0	96,2	93,7	95
72	0	104,8	84,6	95
73	0	95,6	89,9	97
74	0	111	86	92
75	0	117,8	102,9	98
76	0	95,6	69,8	99
77	15	112	53,2	90
78	5	95	60,5	98
79	0	113	82	98
80	0	112,6	75,2	98
81	0	118,9	77,5	98
82	0	103,1	92	93
83	0	109,8	85,7	99
84	0	88	87,1	92
85	0	133,9	64,1	98
86	0	106,2	77,6	92
87	0	95,5	61,7	96

88	0			95
89	0	117	81	99
90	0	102,5	73,3	99
91	0	98	106,7	
92	0	92,5	90,4	94
93	1	94,3	65	90
94	13	101,5	80,2	96
95	0	116	82,4	
96	3	86,1	45,8	90
97	0	103,5	96,4	95
98	22	90,3	77,5	93
99	0	94,6	86,3	95
100	0	100,3	85	98
101	0	130,2	83	97
102	0	107	75,9	93
103	0	118,5	73,5	98
104	0	83,1	44,8	95
105	0	107,7	78,2	95
106	8	72,9	77,5	93
107	0	99,4	80,5	93
108	0	111,4	5,67	93
109	0	88,4	87	99
110	0			92
111	0	71,9	48,3	90
112	0	84,7	70,6	93
113	4	87,5	64,4	98
114	0	104,4	83,1	94
115	0	85	66,8	83
116	0	103,2	89,7	97
117	0	100,5	70,9	83

118	10	73,2	53,7	88
119	0	89,6	79,4	99
120	0	98,7	83,8	93
121	0	113	68	97
122	0	124,9	94,3	96
123	0	116,4	87,3	99
124	17	87,9	38,3	88
125	12	97,4	75,3	90
126	10	101,5	86	97
127	6	116,7	66,2	95
128	9	71,9	39,9	91
129	8	86,4	63,5	
130	0	92,5	70,5	88
131	0	100,9	83,1	98
132	0	89,6	73,6	96
133	0	86,2	60,7	97
134	0	95,4	83,2	97
135	10	115,2	85,7	93
136	0	99,3	104,5	93
137	11	102,1	82,7	94
138	0	111,7	75,3	94
139	9	88,3	73,5	94
140	0	97,7	65,3	94
141	0	85,1	88,4	
142	7	126	83	96
143	6	34,2	33,5	97
144	0	114,2	68,9	96
145	0	97,3	88,2	94
146	0	133,2	74,5	95
147	7	98	71	95

148	0	115,9	86,9	94
149	0	114	97,7	99
150	0	96,1	109,3	93
151	0	84	100,4	90
152	0	114	55,6	95
153	4	108,3	60,7	94
154	0	116,6	94,5	95
155	3	100,5	84,3	92
156	0	95	69,2	94
157	13	93,3	31,7	
158	0	116,4	65,7	95
159	0	112,8	76,6	78
160	13	120,9	69,3	93
161	0	92,6	67,7	86
162	0	93,3	93,9	97
163	12	108,3	76,1	92
164	0	105,6	92,1	94
165	0	100,8	74,5	98
166	5	90,5	75,4	99
167	0	122,2	92,4	97
168	0	107,3	83,5	96
169	11	92,3	56,3	99
170	0	83,8	64,6	94
171	0	92,7	72,4	93
172	11	84,9	58,4	89
173	0	123	82	98
174	11	88	76,8	91
175	0	110,8	73,2	97
176	10	107,3	92,1	93
177	0	90,6	77,3	96

178	0	137,5	101,7	88
179	3	86,5	66,6	94
180	0	81,3	91,5	99
181	7	103	64	93
182	12	77,7	78,7	90
183	0	114,4	6,17	98
184	0	75,3	60,8	95
185	0	118	96,1	96
186	0	98	57,4	98
187	0	99,3	102,2	97
188	0	65,1	60,7	95
189	0	102,8	80,1	99
190	0	88,2	63,3	91
191	9	93,5	70,1	
192	0	130	79,3	98
193	0	91,2	73,8	95
194	0	93,8	96,5	93
195	3	67	54	86
196	0	107,9	101	98
197	0	128	89	97
198	0	99,9	82	97
199	0	141,1	91,1	98
200	0	87,8	71,7	92
201	8	120	66	96
202	0	108,9	68,1	91
203	0	105,9	81,5	95
204	0	88,3	88,6	
205	0	100,4	101,5	93
206	0	69,3	61,9	92
207	13	74,1	60,1	85

208	0	100,6	83,6	90
209	0	114,8	88,2	95
210	0	98,9	75,4	97
211	0	87,6	72,8	98
212	0	121	86,4	96
213	0	113,3	93	94
214	0	110,2	59,6	92
215	0	106,2	73,2	94
216	0	93,4	70,7	95
217	6	95,2	85,3	96
218	0	119,3	94,7	98
219	0	110	61	99
220	0	81,4	60,1	98
221	27	56	38	82
222	0	77,8	79,6	94
223	0	107,1	95,9	95
224	0			99
225	0	114,5	96,4	95
226	0	100,8	77,6	95
227	0	105,5	67,6	85
228	0	60,5	66,4	93
229	0	77,5	94,1	93
230	6	84	100,8	96
231	11	101,8	63,4	97
232	15	65,8	87	
233	0	94,1	92,2	96
234	0	101,8	74,5	96
235	15	101,9	94,6	98
236	0	122,8	85,2	95
237	0	117	76	76

238	0	141,6	109,7	98
239	0	106,1	90,1	95
240	0	113,3	62,4	98
241	0	86	50,3	
242	0	92,2	70	98
243	0	76,9	85,8	93
244	0	135,3	71,8	99
245	0	110,7	61,2	85
246	5	100,8	83,9	94
247	0	106,9	102,9	91
248	0	127,5	85,1	95
249	0	119,6	81	91
250	0	95,4	94	90
251	57	95,4	40,9	90
252	0	90,4	65,2	89
