# Supplementary material for: Prevalence and Cardiopulmonary Characteristics of Post-COVID Syndrome at a Hungarian Tertiary Referral Hospital
Source: J Clin Med. 2025 Apr 10;14(8):2604. doi: 10.3390/jcm14082604 (PMC12028108; doi:10.3390/jcm14082604)
Supplement: Supplementary file 1 [file jcm-14-02604-s001.zip › S8_Supporting information for Figure 4.pdf]

Figure S8. Supporting information for Figure 4.

Participant	Length of hospital stay (days)	NT-proBNP (pg/ml)	Troponin-T (ng/l)
1	0	167	3
2	0	78	16,81
3	22	89	4,94
4	7	90	6,95
5	9	62	7,84
6	0	27	8,18
7	0	50	4,7
8	0	27	5,42
9	0	41	11,6
10	8	59	5
11	12	64	6,76
12	0	19	3
13	6	162	32,91
14	7	180	7,72
15	16	122	10,2
16	0	61	3
17	0	34	3
18	0	10	3
19	8	262	11,32
20	0	45	5,9
21	0	488	7,18
22	0	39	12,02
23	0	22	4,27
24	0	10	6,97
25	0	10	3,36
26	7	181	5,89
27	0	86	6,25

28	7	95	3
29	19	136	16,05
30	0	143	3,68
31	0	131	4,59
32	0	33	3,33
33	0	31	3
34	0	18	6,16
35	0	78	6,86
36	0	24	5,99
37	0	33	4,2
38	0	30	6,99
39	12	200	16,71
40	0	10	3,57
41	13	63	7,93
42	0	280	5,55
43	0	77	6,81
44	15	597	7,96
45	11	51	7
46	0	151	7,44
47	0	16	6,66
48	0	120	3,29
49	0	39	3
50	1	30	3,6
51	0	85	3
52	0	78	3
53	0	45	3,68
54	0	99	5,08
55	0	16	3
56	10	197	34,75
57	0	102	30,07

58	5	247	9,91
59	7	370	19,64
60	8	31	4,39
61	0	161	4,32
62	0	123	3,22
63	17	294	15,46
64	6	348	10,07
65	0	46	3
66	9	19	5,8
67	0	33	5,36
68	0	48	3
69	15	23	10,28
70	0	20	3
71	0	18	6,58
72	0	41	5,68
73	0	58	5,09
74	0	142	8,58
75	0	42	3,54
76	0	40	4,02
77	15	85	7,63
78	5	114	6,88
79	0	47	3
80	0	119	3
81	0	32	6,93
82	0	27	3,31
83	0	139	3
84	0	536	3,43
85	0	56	9,63
86	0	128	5,86
87	0	10	3

88	0	10	7,3
89	0	36	3,69
90	0	41	3
91	0	10	3
92	0	21	4,48
93	1	75	14,56
94	13	10	3
95	0	28	4,38
96	3	111	24,6
97	0	47	6,62
98	22	80	16,72
99	0	45	5,71
100	0	17	11
101	0	73	3
102	0	144	3,92
103	0	40	4,05
104	0	443	13,7
105	0	125	7,66
106	8	64	4,93
107	0	43	10,71
108	0	24	3
109	0	121	4,83
110	0	54	12,22
111	0	2003	21,8
112	0	10	6,84
113	4	141	4,24
114	0	23	3
115	0	550	5,46
116	0	76	3
117	0	38	7,01

118	10	254	21,27
119	0	275	3
120	0	21	3
121	0	390	7,26
122	0	30	3
123	0	98	3,92
124	17	152	9
125	12	182	16,13
126	10	75	15,6
127	6	43	4,3
128	9	74	13,43
129	8	240	7,53
130	0	38	3
131	0	42	3
132	0	45	3
133	0	186	3
134	0	29	3
135	10	371	7,02
136	0	58	3,35
137	11	94	5,67
138	0	122	11
139	9	35	11,46
140	0	14	3
141	0	97	25,3
142	7	28	3,63
143	6	129	13,34
144	0	79	4,2
145	0	322	4,66
146	0	212	11,06
147	7	30	5,84

148	0	82	3
149	0	10	5,61
150	0	25	3
151	0	53	5,92
152	0	77	3
153	4	10	3
154	0	305	16,23
155	3	30	4,17
156	0	69	4,68
157	13	108	3,45
158	0	116	5,36
159	0	214	6,55
160	13	29	11,57
161	0	194	3
162	0	76	7,77
163	12	86	4,13
164	0	29	6,49
165	0	52	3,23
166	5	45	7,4
167	0	38	6,14
168	0	68	3
169	11	35	3
170	0	99	17,37
171	0	61	5,96
172	11	123	8,89
173	0	47	3
174	11	275	11,7
175	0	58	7,26
176	10	81	7,28
177	0	13	3

178	0	41	11,5
179	3	885	14,34
180	0	32	3,4
181	7	27	11,34
182	12	71	17,92
183	0	154	11,92
184	0	113	4,59
185	0	31	5,8
186	0	98	6,55
187	0	21	4,09
188	0	24	3,57
189	0	131	4,6
190	0		7,2
191	9	233	7,16
192	0	39	3,32
193	0	10	3,67
194	0	121	27,25
195	3	201	21,04
196	0	29	7,19
197	0	120	3
198	0	152	11,86
199	0	64	9,46
200	0	285	6,95
201	8	19	4,13
202	0	202	10,24
203	0	16	5,55
204	0	18	3,28
205	0	44	6,88
206	0	41	12,97
207	13	70	17,14

208	0	98	3,46
209	0	98	4,9
210	0	18	5,35
211	0	51	3
212	0	37	3,09
213	0	31	3
214	0	142	6,65
215	0	92	
216	0	118	3
217	6	20	3
218	0	79	3,52
219	0	35	4,53
220	0	175	3,6
221	27	198	13,1
222	0	27	5,36
223	0	24	3,8
224	0	32	3
225	0	130	6,02
226	0	116	4,78
227	0	96	3,76
228	0	84	4,13
229	0	39	3,45
230	6	10	4,92
231	11	469	3
232	15	94	15,94
233	0	63	22,77
234	0	35	8,05
235	15	29	4,58
236	0	30	4,24
237	0	14	3

238	0	78	13,34
239	0	70	14,13
240	0	95	4,84
241	0	72	3,05
242	0	210	5,4
243	0	176	3,9
244	0	175	8,26
245	0	251	176,4
246	5	10	6,37
247	0	28	6,33
248	0	41	3
249	0	18	3
250	0	72	8,84
251	57	765	35,64
252	0	62	6,42
