# Supplementary material for: Prevalence and Cardiopulmonary Characteristics of Post-COVID Syndrome at a Hungarian Tertiary Referral Hospital
Source: J Clin Med. 2025 Apr 10;14(8):2604. doi: 10.3390/jcm14082604 (PMC12028108; doi:10.3390/jcm14082604)
Supplement: Supplementary file 1 [file jcm-14-02604-s001.zip › S9_Supporting information for Table 5 and Figure 5.pdf]

Figure S9. Supporting information for Table 5 and Figure 5.

Participant	Age	Infection severity	Length of hospital stay (days)
1	46	Treated at home	0
2	63	Treated at home	0
3	65	Hospitalized with respiratory failure	22
4	55	Hospitalized with respiratory failure	7
5	54	Hospitalized with respiratory failure	9
6	37	Treated at home	0
7	58	Treated at home	0
8	40	Treated at home	0
9	61	Treated at home	0
10	45	Hospitalized with respiratory failure	8
11	54	Hospitalized with respiratory failure	12
12	28	Treated at home	0
13	63	Hospitalized with respiratory failure	6
14	67	Hospitalized with respiratory failure	7
15	62	Hospitalized with respiratory failure	16
16	32	Treated at home	0
17	45	Treated at home	0
18	38	Treated at home	0
19	72	Required intensive care	8
20	52	Treated at home	0
21	65	Treated at home	0
22	65	Hospitalized with respiratory failure	0
23	36	Treated at home	0
24	51	Treated at home	0
25	21	Treated at home	0
26	44	Hospitalized with respiratory failure	7
27	39	Treated at home	0

28	45	Hospitalized without respiratory failure	7
29	73	Hospitalized without respiratory failure	19
30	47	Treated at home	0
31	66	Hospitalized with respiratory failure	0
32	41	Treated at home	0
33	40	Treated at home	0
34	57	Treated at home	0
35	48	Treated at home	0
36	60	Treated at home	0
37	46	Treated at home	0
38	48	Treated at home	0
39	56	Hospitalized with respiratory failure	12
40	33	Treated at home	0
41	68	Hospitalized with respiratory failure	13
42	60	Treated at home	0
43	65	Treated at home	0
44	66	Hospitalized with respiratory failure	15
45	65	Required intensive care	11
46	60	Treated at home	0
47	62	Treated at home	0
48	50	Treated at home	0
49	38	Treated at home	0
50	45	Hospitalized without respiratory failure	1
51	44	Treated at home	0
52	46	Treated at home	0
53	47	Treated at home	0
54	26	Treated at home	0
55	32	Treated at home	0
56	85	Hospitalized without respiratory failure	10
57	69	Treated at home	0

58	66	Hospitalized without respiratory failure	5
59	63	Hospitalized with respiratory failure	7
60	65	Hospitalized with respiratory failure	8
61	49	Treated at home	0
62	77	Treated at home	0
63	79	Hospitalized with respiratory failure	17
64	58	Hospitalized with respiratory failure	6
65	67	Treated at home	0
66	60	Hospitalized with respiratory failure	9
67	55	Treated at home	0
68	38	Treated at home	0
69	48	Hospitalized with respiratory failure	15
70	44	Treated at home	0
71	61	Treated at home	0
72	54	Treated at home	0
73	47	Treated at home	0
74	64	Treated at home	0
75	37	Treated at home	0
76	43	Treated at home	0
77	65	Hospitalized without respiratory failure	15
78	66	Hospitalized with respiratory failure	5
79	51	Treated at home	0
80	61	Treated at home	0
81	39	Treated at home	0
82	66	Treated at home	0
83	20	Treated at home	0
84	55	Treated at home	0
85	77	Treated at home	0
86	48	Treated at home	0
87	54	Treated at home	0

88	46	Treated at home	0
89	32	Treated at home	0
90	30	Treated at home	0
91	42	Treated at home	0
92	28	Treated at home	0
93	53	Required intensive care	1
94	38	Hospitalized with respiratory failure	13
95	50	Treated at home	0
96	56	Required intensive care	3
97	68	Treated at home	0
98	71	Hospitalized with respiratory failure	22
99	36	Treated at home	0
100	27	Treated at home	0
101	24	Treated at home	0
102	47	Treated at home	0
103	41	Treated at home	0
104	78	Treated at home	0
105	79	Treated at home	0
106	39	Hospitalized with respiratory failure	8
107	42	Treated at home	0
108	56	Treated at home	0
109	42	Treated at home	0
110	74	Treated at home	0
111	71	Treated at home	0
112	43	Treated at home	0
113	47	Required intensive care	4
114	48	Treated at home	0
115	62	Treated at home	0
116	38	Treated at home	0
117	45	Treated at home	0

118	64	Hospitalized with respiratory failure	10
119	33	Treated at home	0
120	42	Treated at home	0
121	65	Treated at home	0
122	21	Treated at home	0
123	50	Treated at home	0
124	70	Hospitalized with respiratory failure	17
125	76	Required intensive care	12
126	39	Hospitalized with respiratory failure	10
127	67	Hospitalized with respiratory failure	6
128	55	Hospitalized with respiratory failure	9
129	59	Hospitalized with respiratory failure	8
130	25	Treated at home	0
131	37	Treated at home	0
132	47	Hospitalized without respiratory failure	0
133	67	Treated at home	0
134	42	Treated at home	0
135	59	Hospitalized with respiratory failure	10
136	54	Treated at home	0
137	50	Hospitalized with respiratory failure	11
138	67	Treated at home	0
139	57	Hospitalized with respiratory failure	9
140	44	Treated at home	0
141	72	Treated at home	0
142	51	Hospitalized without respiratory failure	7
143	61	Required intensive care	6
144	33	Treated at home	0
145	46	Treated at home	0
146	72	Treated at home	0
147	47	Hospitalized with respiratory failure	7

148	64	Treated at home	0
149	22	Treated at home	0
150	48	Treated at home	0
151	60	Treated at home	0
152	46	Treated at home	0
153	36	Hospitalized without respiratory failure	4
154	81	Treated at home	0
155	52	Hospitalized with respiratory failure	3
156	34	Treated at home	0
157	65	Hospitalized with respiratory failure	13
158	47	Treated at home	0
159	41	Treated at home	0
160	67	Hospitalized with respiratory failure	13
161	43	Treated at home	0
162	50	Treated at home	0
163	54	Hospitalized with respiratory failure	12
164	46	Treated at home	0
165	38	Treated at home	0
166	44	Hospitalized without respiratory failure	5
167	64	Treated at home	0
168	45	Treated at home	0
169	53	Hospitalized with respiratory failure	11
170	49	Treated at home	0
171	50	Treated at home	0
172	59	Required intensive care	11
173	36	Treated at home	0
174	70	Hospitalized with respiratory failure	11
175	66	Treated at home	0
176	66	Hospitalized with respiratory failure	10
177	37	Treated at home	0

178	62	Treated at home	0
179	89	Required intensive care	3
180	29	Treated at home	0
181	54	Hospitalized with respiratory failure	7
182	72	Hospitalized with respiratory failure	12
183	52	Treated at home	0
184	54	Treated at home	0
185	47	Treated at home	0
186	55	Treated at home	0
187	47	Treated at home	0
188	47	Treated at home	0
189	60	Treated at home	0
190	65	Treated at home	0
191	65	Hospitalized with respiratory failure	9
192	60	Treated at home	0
193	42	Treated at home	0
194	60	Hospitalized with respiratory failure	0
195	57	Hospitalized with respiratory failure	3
196	59	Treated at home	0
197	39	Treated at home	0
198	59	Treated at home	0
199	56	Treated at home	0
200	64	Treated at home	0
201	48	Hospitalized without respiratory failure	8
202	59	Treated at home	0
203	56	Treated at home	0
204	38	Treated at home	0
205	54	Treated at home	0
206	53	Treated at home	0
207	65	Required intensive care	13

208	38	Treated at home	0
209	65	Treated at home	0
210	27	Treated at home	0
211	31	Treated at home	0
212	58	Treated at home	0
213	49	Treated at home	0
214	55	Treated at home	0
215	48	Treated at home	0
216	52	Treated at home	0
217	53	Hospitalized without respiratory failure	6
218	45	Treated at home	0
219	33	Treated at home	0
220	70	Hospitalized with respiratory failure	0
221	62	Required intensive care	27
222	52	Treated at home	0
223	31	Treated at home	0
224	31	Treated at home	0
225	60	Treated at home	0
226	68	Treated at home	0
227	49	Treated at home	0
228	57	Treated at home	0
229	20	Treated at home	0
230	47	Hospitalized without respiratory failure	6
231	57	Hospitalized with respiratory failure	11
232	42	Required intensive care	15
233	35	Treated at home	0
234	22	Treated at home	0
235	21	Required intensive care	15
236	48	Treated at home	0
237	47	Treated at home	0

238	62	Treated at home	0
239	65	Treated at home	0
240	31	Treated at home	0
241	65	Treated at home	0
242	58	Treated at home	0
243	60	Treated at home	0
244	75	Treated at home	0
245	87	Treated at home	0
246	61	Hospitalized with respiratory failure	5
247	52	Treated at home	0
248	53	Treated at home	0
249	45	Treated at home	0
250	64	Treated at home	0
251	80	Hospitalized with respiratory failure	57
252	29	Treated at home	0
